# Genome-wide characterization of *JASMONATE-ZIM DOMAIN* transcription repressors in wheat (*Triticum aestivum* L.)

**DOI:** 10.1186/s12864-017-3582-0

**Published:** 2017-02-13

**Authors:** Yukun Wang, Linyi Qiao, Jianfang Bai, Peng Wang, Wenjing Duan, Shaohua Yuan, Guoliang Yuan, Fengting Zhang, Liping Zhang, Changping Zhao

**Affiliations:** 1 Beijing Engineering Research Center for Hybrid Wheat, Beijing Academy of Agricultural and Forestry Sciences, Beijing, 100097 China; 2 The Municipal Key Laboratory of Molecular Genetics for Hybrid Wheat, Beijing, 100097 China; 3 Institute of Crop Science, Shanxi Academy of Agricultural Sciences, Taiyuan, 030031 China; 4 College of Plant Science and Technology, Beijing University of Agriculture, Beijing, 102206 China; 5 College of Life Sciences, Capital Normal University, Beijing, 100048 China

**Keywords:** Anther dehiscence, Gene expression, JASMONATE-ZIM DOMAIN, Phylogenetic analysis, Thermo sensitive genic male sterile (TGMS)

## Abstract

**Background:**

The JASMONATE-ZIM DOMAIN (JAZ) repressor family proteins are jasmonate co-receptors and transcriptional repressor in jasmonic acid (JA) signaling pathway, and they play important roles in regulating the growth and development of plants. Recently, more and more researches on *JAZ* gene family are reported in many plants. Although the genome sequencing of common wheat (*Triticum aestivum* L.) and its relatives is complete, our knowledge about this gene family remains vacant.

**Results:**

Fourteen *JAZ* genes were identified in the wheat genome. Structural analysis revealed that the TaJAZ proteins in wheat were as conserved as those in other plants, but had structural characteristics. By phylogenetic analysis, all JAZ proteins from wheat and other plants were clustered into 11 sub-groups (G1-G11), and TaJAZ proteins shared a high degree of similarity with some JAZ proteins from *Aegliops tauschii*, *Brachypodium distachyon* and *Oryza sativa*. The Ka/Ks ratios of *TaJAZ* genes ranged from 0.0016 to 0.6973, suggesting that the *TaJAZ* family had undergone purifying selection in wheat. Gene expression patterns obtained by quantitative real-time PCR (qRT-PCR) revealed differential temporal and spatial regulation of *TaJAZ* genes under multifarious abiotic stress treatments of high salinity, drought, cold and phytohormone. Among these, *TaJAZ7*, *8* and *12* were specifically expressed in the anther tissues of the thermosensitive genic male sterile (TGMS) wheat line BS366 and normal control wheat line Jing411. Compared with the gene expression patterns in the normal wheat line Jing411, *TaJAZ7*, *8* and *12* had different expression patterns in abnormally dehiscent anthers of BS366 at the heading stage 6, suggesting that specific up- or down-regulation of these genes might be associated with the abnormal anther dehiscence in TGMS wheat line.

**Conclusion:**

This study analyzed the size and composition of the *JAZ* gene family in wheat, and investigated stress responsive and differential tissue-specific expression profiles of each *TaJAZ* gene in TGMS wheat line BS366. In addition, we isolated 3 *TaJAZ* genes that would be more likely to be involved in the regulation of abnormal anther dehiscence in TGMS wheat line. In conclusion, the results of this study contributed some novel and detailed information about *JAZ* gene family in wheat, and also provided 3 potential candidate genes for improving the TGMS wheat line.

**Electronic supplementary material:**

The online version of this article (doi:10.1186/s12864-017-3582-0) contains supplementary material, which is available to authorized users.

## Background

Jasmonic acid and its bioactivity derivatives are collectively known as Jasmonates (JAs). They play important roles in regulating the responses to biotic and abiotic stresses, such as herbivory, pathogen invasion, wounding, UV radiation and ozone stress in plants [1–8]. In addition, JAs also control various developmental processes in plants, viz. pollen maturation, anther dehiscence, embryo maturation, tendril coiling, tuber and trichome growth [9–12]. Therefore, JAs are regarded as moderators that mainly involved in regulating the defense reaction, inhibiting the photosynthesis and cell division, and keeping the balance between growth and defense in plants [2, 13, 14].

In plants, JA signaling pathway is a very complex process involved in many genes or proteins [15, 16], and this pathway can be divided into three processes, including the biosynthesis and metabolism of signaling molecules, JA signal transduction and responses of downstream genes. Previous studies showed that JA signaling molecules, SCF^COI1^ receptor complex, Jasmonate-ZIM (JAZ) domain repressor and transcription activator MYC2 participate together and interact with each other in JA signaling pathway [17–21]. In the plant cells with a lower-concentration of JA, the transcription of JA response genes is restrained owing to the combination between JAZ protein and MYC2. Under stimulation, JA is compounded and accumulated in plant cells. High concentrations of JA can promote the combination between JAZ proteins and SCF^COI1^, and make JAZ proteins be ubiquitinated. Then the ubiquitinated protein is alternatively degraded by 26S protease. Finally, the activity inhibition of transcription activator MYC2 is removed. Expression of JA response genes is activated subsequently [19]. It is known that JAZ proteins have a crucial effect on the JA signaling pathway and they are the linkers of JA signaling transduction.

The JAZ protein family is a member of TIFY transcription factor superfamily, and the family members have two conservative functional domains, TIFY (also known as ZIM) and Jas (also known as CCT_2) [22–24]. TIFY domain is usually consisted of 28 amino acids, which located in the N-terminus of JAZ protein sequences, and its core sequence is TIF[F/Y]XG [25, 26]. In JA signaling pathway, TIFY domain can mediate the interaction between JAZ protein and its co-suppressor, a NOVEL INTERACTOR of JAZ (NINJA), and then collectively restrict the JA signal transduction [27]. Jas domain is near the C-terminus of JAZ protein sequences, and its sequence is extremely conserved among the members of JAZ protein family, of which 10 amino acids are same or replaced in conservation. Its function is to mediate the direct combination between JAZ protein and MYC2 in JA signaling pathway and inhibit the transcriptional activity of MYC2 [28, 29], and then further restrict the expression of JA response genes.

As the connector between MYC2 and COI1, JAZ protein was firstly reported in 2007 [30, 31]. Afterwards, many studies about JAZ proteins have been conducted. To date, 12 and 15 *JAZ* genes have been found in *Arabidopsis thaliana* [13] and rice [32], respectively. According to previous studies, the *JAZ* gene family has plenty of members, but each has a different biological function. For example, the protein encoded by *OsJAZ9* is a regulator of JA signaling pathway and can regulate the salt tolerance of rice [33]. The expression of *GhJAZ1* can be activated by *GbWRKY1*, so that it can strengthen the capacity for resistance against *Verticillium dahlia* in cotton [34]. Overexpression of modified *AtJAZ1* (lack of Jas domain) is able to enhance host resistance to *Spodoptera exigua* in Arabidopsis [35]. Overexpressing the modified *OsJAZs* can cause the malformation of flower organ in rice [36]. According to the above researches, *JAZ* genes have important effects on regulating the adaptability to biotic and abiotic stresses and maintaining the normal development in plants.

Wheat is one of the most important crops in the world. The changes of climate and growing environments bring huge challenge to wheat production. Thus, use of molecular biology and genetics methods is an important approach for improving the stress tolerance and quality of wheat. Thermosensitive genic male sterile (TGMS) lines, such as BS366, are of particular significance in two-line hybrid system, which is more efficient in breeding [37], because the fertility of TGMS lines is strictly regulated by temperature [38–40]. Hybrid seed could be produced under sterile condition (TGMS line as maternal plant), while TGMS line itself could be reproduced under fertile condition [41]. In our early research, we found that the anther dehiscence in BS366 is abnormal, and its pollen can not fully spill out. This defective phenotype can be recovered via spraying MeJA in vitro [42]. So, we assumed that this phenotype is closely related to the synthesis and regulatory pathway of JA. Based on the current wheat genome sequencing data, we firstly identified the *TaJAZ* gene family, and analyzed its architectural features, evolutionary history and expression patterns in anther tissues in TGMS wheat line. The results presented in this study were expected to enrich our knowledge of *JAZ* gene family in wheat, and provide the theoretical basis and novel candidate genes for improving and creating the male sterility wheat lines.

## Results

### Identification of JAZ repressor gene family in wheat

**Fig. 1:**
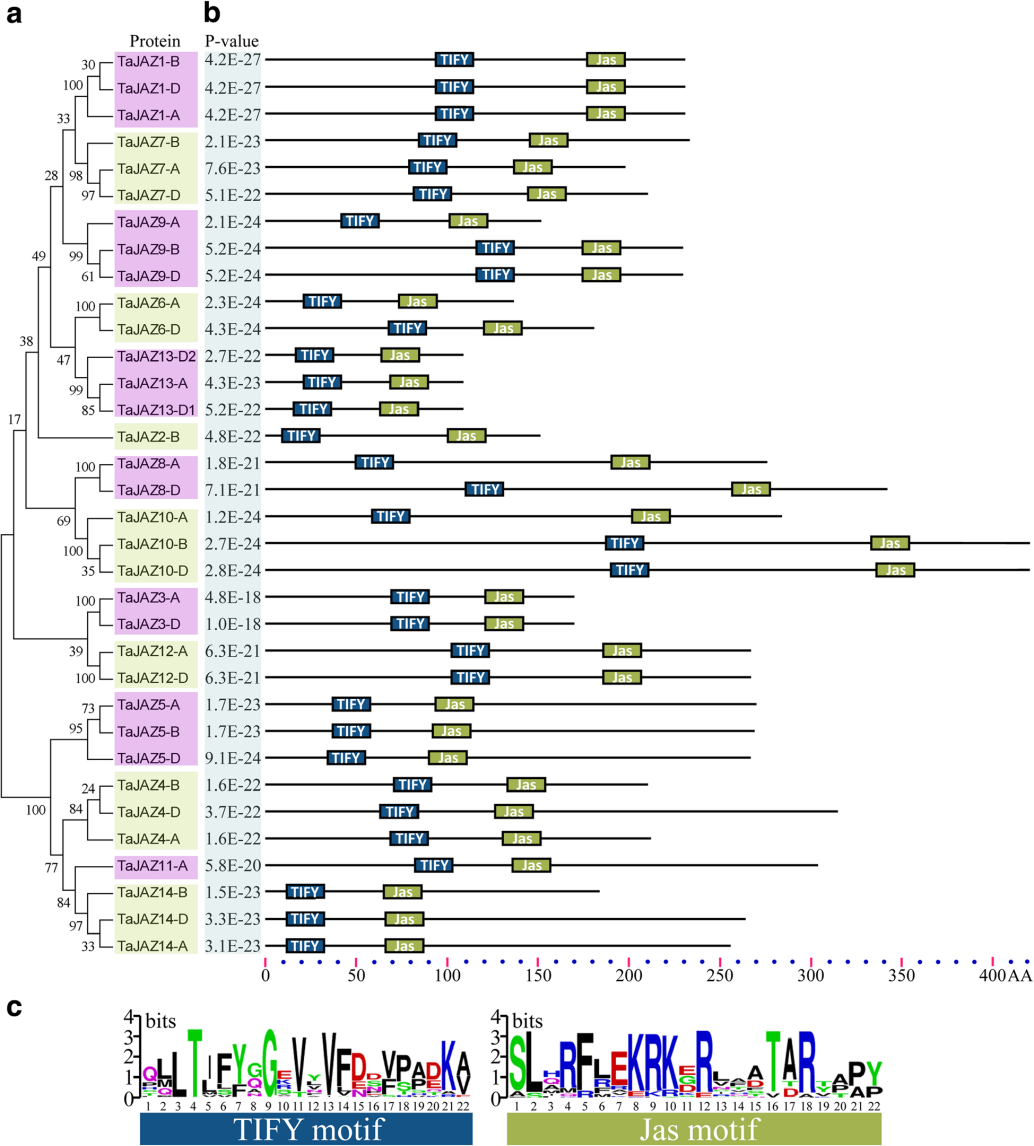
Phylogenetic relationship and motif structure of wheat JAZ proteins. **a** The phylogenetic tree of TaJAZ proteins constructed from a complete alignment of 34 wheat JAZ proteins using MEGA 6.0 by the neighbor-joining method with 1000 bootstrap replicates. Bootstrap scores are indicated on the nodes and the 14 members of TaJAZ, most of which contain duplicated genes, are indicated by yellow or pink block. **b** Domain distribution of TaJAZ proteins were investigated using the MEME web server. Color blocks represent the position of motifs on corresponding proteins. **c** The consensus sequence of TIFY and Jas motif from wheat JAZ proteins. The relative position of each motif can be determined using the scale below

Based on the latest genome data of wheat, a hidden Markov model (HMM) search was carried out using the HMM profiles of the TIFY domain (Pfam accession No.: PF06200) and Jas domain (Pfam accession No.: PF09425) as queries against the local protein database. By retrieving the database, we detected 40 non-redundant sequences. Among these, 6 were removed because they lacked the typical TIFY or Jas domain. Finally, we obtained 34 full-length protein sequences (Table 1). The corresponding coding sequences and genome sequences were simultaneously isolated. The location of each *TaJAZ* gene was confirmed by aligning the wheat chromosome genomic sequences using BLASTn. According to the information of phylogenetic relationship, 34 *TaJAZ* genes were clustered into 14 groups (Fig. 1a). The genes from different wheat sub-genomes and in the same group were regarded as different copies of each member of the *TaJAZ* gene family. Thus, we obtained the *TaJAZ* gene family including 14 members designated from 1 to 14 according to the naming convention, and the copies of each member were distinguished by subjoining the wheat sub-genome symbols A, B or D.

**Table 1:** *JAZ* gene family in wheat

Genes	Sequence ID^a^	Scaffold	Location	Length gDNA	Length AA
*TaJAZ1-A*	Traes_2AS_A8CCC32D3.1	5193315	2AS:194518416-194520896	1657	232
*TaJAZ1-B*	Traes_2BS_2C79AE2DE.1	5245909	2BS:116670615-116671727	1628	232
*TaJAZ1-D*	Traes_2DS_C0C75D1D7.1	5342634	2DS:43488169-43490434	1645	232
*TaJAZ2-B*	Traes_2BL_0614A2B97.2	8023225	2BL:325113849-325118711	1568	152
*TaJAZ3-A*	Traes_2AL_6CBE19B87.2	6411971	2AL:249758979-249759677	3163	171
*TaJAZ3-D*	Traes_2DL_7DD4A39D4.2	9853109	2DL:75977386-75980215	2180	171
*TaJAZ4-A*	Traes_4AL_406C57F67.2	7068037	4AL:117374944-117379369	2615	213
*TaJAZ4-B*	Traes_4BS_8C20E76AA.2	4872575	4BS:8921165-8925871	2851	211
*TaJAZ4-D*	Traes_4DS_AFCEDDE67.2	2320697	4DS:39879093-39878219	4174	316
*TaJAZ5-A*	Traes_4AL_B6992AAA6.1	7095962	4AL:153112902-153117061	3902	271
*TaJAZ5-B*	Traes_4BS_ACD70539F.1	4890614	4BS:208712283-208716179	3616	270
*TaJAZ5-D*	Ta4dsLoc000428.2	1474230	4DS:5798624-5803047	3875	268
*TaJAZ6-A*	Traes_4AS_6EAA11AAD.2	5953686	4AS:31692130-31692495	539	138
*TaJAZ6-D*	Traes_4DL_E25D3DF01.1	14389161	4DL:102441958-102442530	546	182
*TaJAZ7-A*	Ta4asLoc008691.5	5960531	4AS:46818670-46818870	829	199
*TaJAZ7-B*	Traes_4BL_AA16A6065.2	7038768	4BL:296045108-296046696	932	234
*TaJAZ7-D*	Traes_4DL_7564D43A9.1	14466294	4DL:112478276-112479120	845	211
*TaJAZ8-A*	Ta5alLoc014221.1	2768335	5AL:143197395-143200628	1409	277
*TaJAZ8-D*	Ta4dlLoc004501.1	14305556	4DL:120302264-120302430	1639	343
*TaJAZ9-A*	Traes_5AL_BB55F989A.2	2752881	5AL:106813685-106814577	701	152
*TaJAZ9-B*	Traes_5BL_7A6C3831E.1	10804653	5BL:50144832-50146521	1106	231
*TaJAZ9-D*	Traes_5DL_4186C5347.1	4498422	5DL:76465423-76467215	1096	231
*TaJAZ10-A*	Traes_5AL_BF3D7E764.1	7360	5AL:146353609-146353412	1269	285
*TaJAZ10-B*	Traes_5BL_1F38B9D05.1	10791567	5BL:76985189-76988406	2325	417
*TaJAZ10-D*	Traes_5DL_3A1F8C38E.1	4511779	5DL:80817088-80820136	2297	419
*TaJAZ11-A*	Traes_6AS_4106E8E28.1	4378986	6AS:35541777-35545250	3030	305
*TaJAZ12-A*	Traes_6AL_BC7FB0A99.1	5834167	6AL:183521365-183523426	1489	268
*TaJAZ12-D*	Traes_6DL_7024F5429.1	1374032	6DL:146803355-146805443	1508	268
*TaJAZ13-A*	Ta7asLoc006840.1	4189390	7AS:48401407-48404266	330	110
*TaJAZ13-D1*	Traes_7DS_0B70FFE9A.1	3814476	7DS:31118625-31119606	330	110
*TaJAZ13-D2*	Traes_7DS_C407F8C85.1	3930115	7DS:39462347-39463081	330	110
*TaJAZ14-A*	Traes_7AL_EA6F4FFDE.2	4463173	7AL:143335085-143340919	5523	257
*TaJAZ14-B*	Traes_7BL_7DC689032.1	6631476	7BL:205014054-205016812	2801	183
*TaJAZ14-D*	Traes_7DL_A5ECEDA95.1	3352851	7DL:203031373-203037757	5602	265

^a^The sequence name beginning with Treas and TaLoc means download from the wheat genome Ensembl database and URGL database, respectively

### Genome distribution of wheat *JAZ* genes

**Fig. 2:**
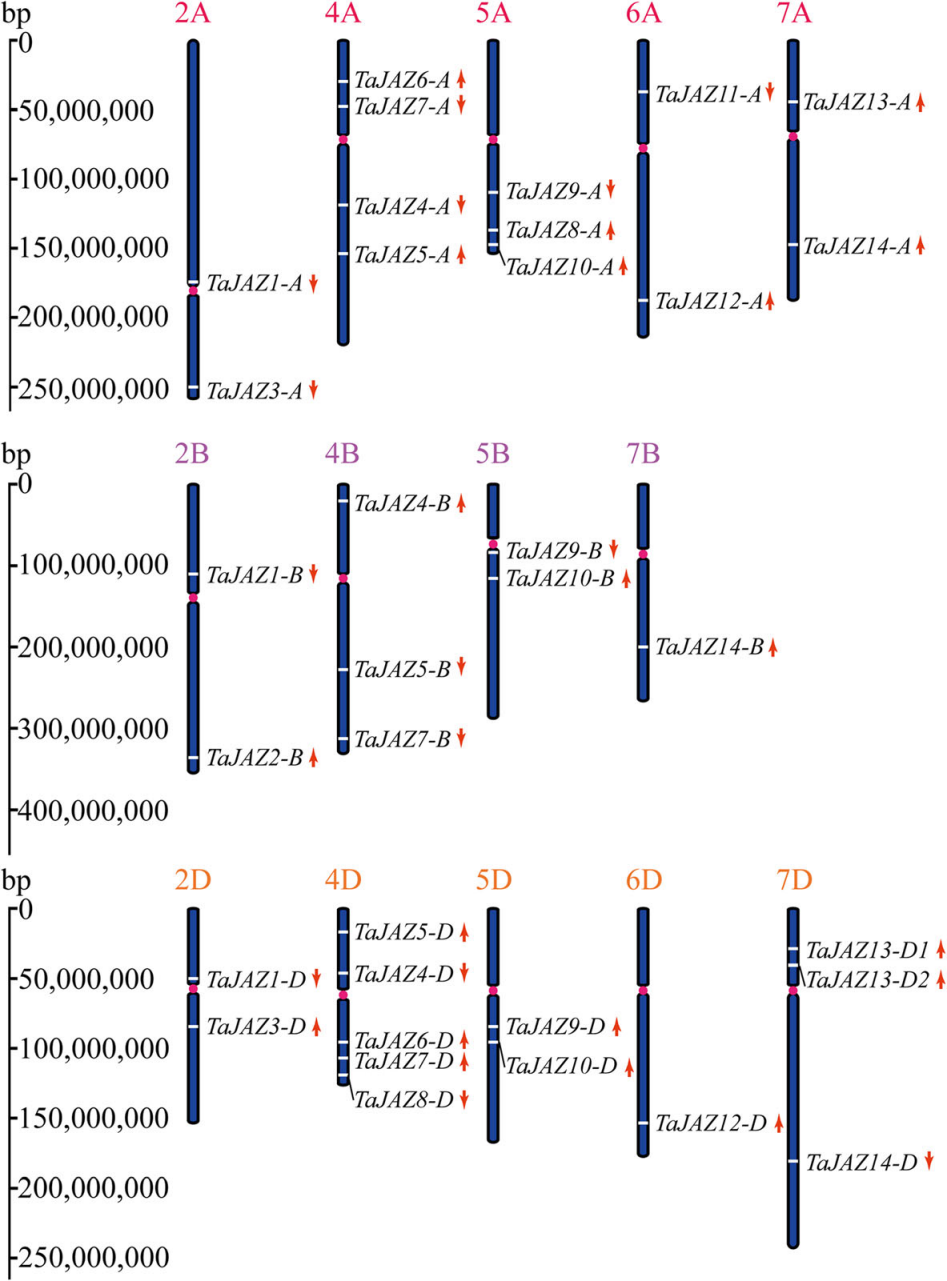
Chromosome distribution of *JAZ* gene family in wheat. Red dots on the chromosomes indicate the position of centromeres. The red arrows next to gene names show the direction of transcription. The position of each gene could be estimated using the left scale

Among 34 wheat *JAZ* genes, 13, 8 and 13 were distributed in wheat sub-genomes A, B and D, respectively (Fig. 2). None of *TaJAZ* gene copy was distributed on chromosomes 1A, 3A, 1B, 3B, 6B, 1D and 3D. Each of the 7 *TaJAZ* genes (*TaJAZ1*, *4*, *5*, *7*, *9*, *10* and *14*) had three copies on group 2, 4, 5 and 7 chromosomes, respectively (Fig. 2). Four *TaJAZ* genes had two copies each including *TaJAZ3-A/-D*, *TaJAZ6-A/-D*, *TaJAZ8-B/-D*, and *TaJAZ12-A/-D* (Fig. 2). *TaJAZ2* and *TaJAZ11* had only one copy on chromosomes 2B and 6A, respectively (Fig. 2). *TaJAZ13* also had three copies, and *TaJAZ13-A* was found on chromosome 7A, *TaJAZ13-D1* and *-D2* were detected on chromosome 7D (Fig. 2). In addition, chromosome 4D had the highest number of five *TaJAZ* gene copies, whereas other chromosomes contained no more than four copies (Fig. 2).

### Structure analysis of wheat *JAZ* genes, and JAZ proteins

**Fig. 3:**
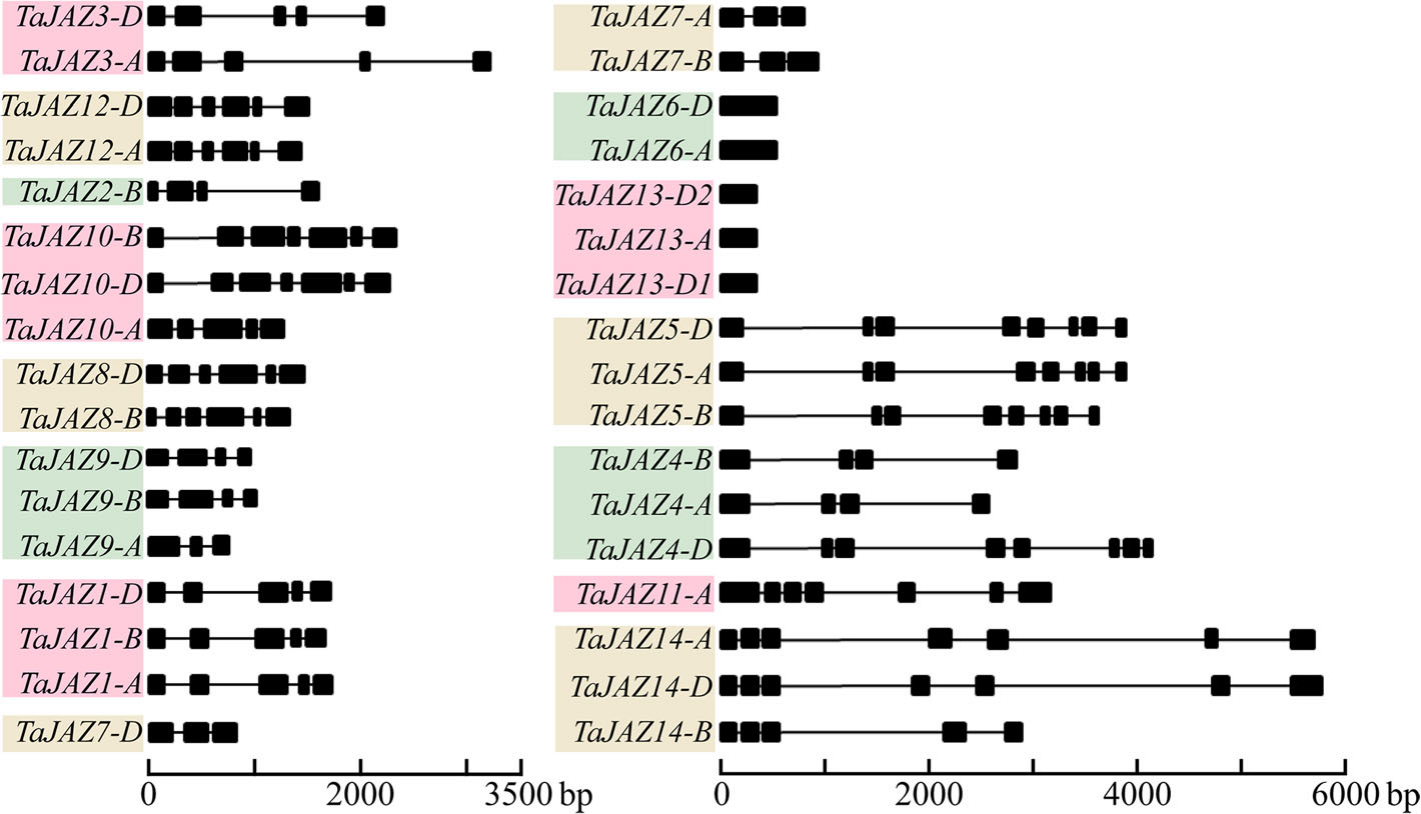
Exon-intron structures of *TaJAZ* genes. Exons are represented by blank boxes and introns by blank lines. The sizes of exons and introns could be estimated using the scale below

In order to obtain more insights about the gene structural evolution, the exon-intron organization of wheat *JAZ* genes was raveled by aligning the predicted coding sequences (CDS) against the corresponding genomic sequences using the online service GSDS. In the *TaJAZ* gene family, the number of exons ranged from 1 to 8, and the number of introns ranged from 0 to 7 (Fig. 3). Among 12 *TaJAZ* genes containing two or three copies, 8 (*TaJAZ1*, *3*, *5*, *6*, *7*, *8*, *12* and *13*) had the same gene structures, whereas 4 (*TaJAZ4*, *9*, *10* and *14*) had one, two or four exons in each group. In addition, *TaJAZ6* and *TaJAZ13* lacked intron (Fig. 3). Overall, a highly similar gene structure was exhibited in the duplicated *TaJAZ* genes.

The TIFY and Jas domains are important for the repressor activity of JAZ proteins in plants. The TIFY and Jas domains locate at the N-terminus and C-terminus of JAZ proteins, respectively. In order to understand the architecture of JAZ proteins in wheat, we scanned these two domains using the MEME web server. As shown in Fig. 1b and c, single TIFY and Jas motifs were distributed in each TaJAZ protein and all TaJAZ proteins had TIFY motif at N-terminus while Jas motif at C-terminus. The results were in accordance with the previous studies [43, 44], indicating that the structures of JAZ proteins were conserved. Moreover, the multi-sequence alignments of protein sequences revealed that the amino acid sequence “TIFY” was changed into “TVFY” in TaJAZ3, “TMFY” in TaJAZ8, “TLFY” in TaJAZ4, “TLVY” in TaJAZ5 and “TLSF” in TaJAZ14 (Additional file 1: Figure S1). The same variations also existed in rice [24], maize [44], and *B. distachyon* [45]. Besides TIFY domain, the Jas domain is also conserved. In Arabidopsis, Jas domain contains 12-29 amino acids, and the sequences are basically coincident or conserved [46]. But in wheat, the Jas domain in some TaJAZ proteins, such as TaJAZ4, 5 and 14, was inserted into a nuclear localization signal (NLS) sequence (Additional file 1: Figure S1).

### Phylogenetic analysis of TaJAZ proteins

**Fig. 4:**
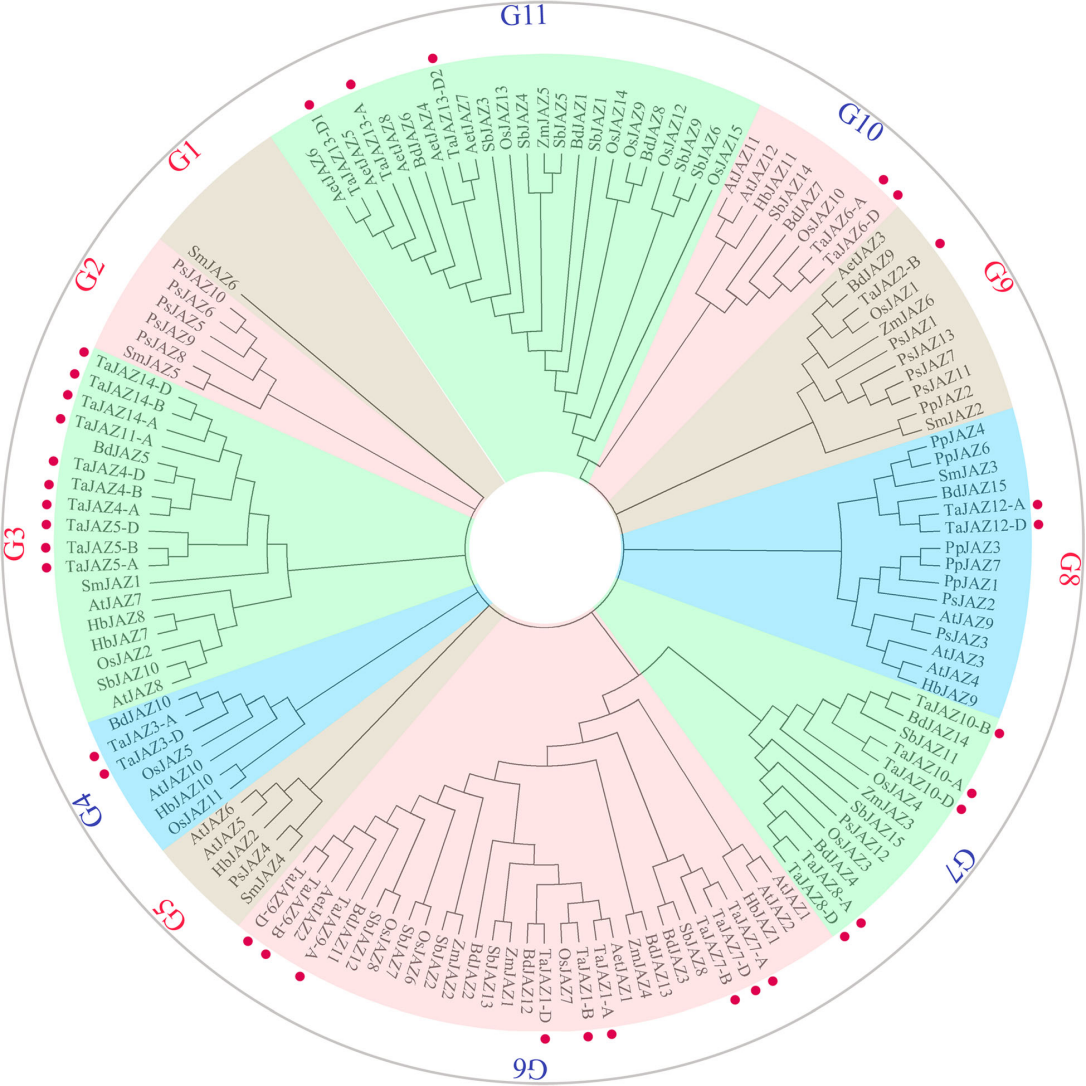
Phylogenetic relationship of JAZ proteins among wheat and other species. The full-length amino acid sequences of 34 *T. aestivum*, 6 *S. moellendorffii*, 12 *A. thaliana*, 15 *O. sativa*, 7 *P. patens*, 6 maize, 15 *B. distachyon*, 15 *S. bicolor*, 13 *P. sitchensis*, 8 *Ae. tauschii* and 7 *H. brasiliensis* genes were aligned by using ClustalX and the phylogenetic tree was constructed using MEGA 6.0 by the neighbor-joining method with 1000 bootstrap replicates. Each TaJAZ protein is indicated by a red dot. Two major groups, group I and II, are represented by the red and blue, respectively

In order to understand the phylogeny of JAZ proteins, N-J tree was built using MEGA 6.0 software and the reliability was tested by bootstrap analysis for 1000 replicates. As shown in Fig. 4, all the JAZ proteins derived from different plants were clustered into sub-groups G1-G11, and the TaJAZ proteins were clustered into sub-groups G3-G11. Sub-group G3 had the maximal number of TaJAZ proteins, and 4 TaJAZ proteins (TaJAZ4, 5, 11 and 14) were clustered into this sub-group. Based on the order that the JAZ proteins appeared in plants, those 11 sub-groups were divided into groups I (marked by red) and II (marked by blue) (Fig. 4). Group I included sub-groups G1, G2, G3, G5, G8 and G9, and group II comprised sub-groups G4, G6, G7, G10 and G11. Moreover, we found that the TaJAZ proteins were clustered into the same clades with some AetJAZ, BdJAZ or OsJAZ proteins. For example, TaJAZ6 was clustered into the sub-group G10 with OsJAZ10, TaJAZ12 was clustered into the sub-group G8 with BdJAZ15, and TaJAZ9 was clustered into the sub-group G6 with AetJAZ2 (Fig. 4). These revealed that the TaJAZ proteins shared a high similarity with those in *Ae. tauschii*, *B. distachyon* and rice.

### Adaptive evolution analysis of the *TaJAZ* gene family

To investigate which type of Darwinian selection determined the process of *TaJAZ* gene divergence after duplication, the Ka/Ks substitution ratio was utilized to the coding sequences of 9 pairs of orthologs between wheat and *B. distachyon JAZ* gene family (Additional file 1: Figure S2). According to the previous research, Ka/Ks ratio < 1 means purifying selection, ratio = 1 means neutral evolution and ratio > 1 means positive selection [47]. As shown in Table 2, the Ka/Ks ratios of *TaJAZ* genes ranged from 0.0016 to 0.6973, suggesting that the *TaJAZ* gene family had undergone purifying selection in wheat.

**Table 2:** Ka/Ks ratio of the duplicated JAZ genes in wheat using *Brachypodium distachyon* as an outgroup

Gene	A-genome	B-genome	D-genome
TaJAZ-1	0.232	0.2405	0.2787
TaJAZ-2	-	0.3099	-
TaJAZ-3	0.3751	-	0.3623
TaJAZ-6	0.0016	-	0.0286
TaJAZ-7	0.2753	0.3521	0.401
TaJAZ-8	0.5066	-	0.6973
TaJAZ-9	0.2887	0.2922	0.2746
TaJAZ-10	0.3754	0.3751	0.3785
TaJAZ-13	0.1222	-	0.2213

### Putative *cis*-acting regulatory elements in the promoter region of *TaJAZ* genes

To gain the information about the *cis*-acting regulatory elements of *TaJAZ* gene family, the putative promoter region sequence of each *TaJAZ* gene was analyzed. In this study, promoter sequences were available for 13 of 14 *TaJAZ* genes, while only the promoter of *TaJAZ11* was unavailable due to the limited genome data of wheat. Eight types of putative cis-acting regulatory elements were identified in *TaJAZ* genes, including LTR element involved in low-temperature responsiveness, TGA element related to Auxin-responsiveness, CGTCA-motif involved in MeJA responsiveness, ABRE associated with ABA signaling pathway, box-w1 for the response of fungal elicitor, GARE-motif of a gibberellin responsive element regulating gibberellin responsive genes, MBS, a MYB-binding sequence involved in regulation of drought-inducible genes and TCA element regulating the SA related genes (Additional file 2: Table S3). The type and number of *cis*-acting regulatory elements in the promoter region of each *TaJAZ* gene were discrepant (Additional file 2: Table S3). These showed that the members of *TaJAZ* gene family might be able to respond to different abiotic stresses.

### Expression profiling of *TaJAZ* genes under abiotic stresses in TGMS wheat line

**Fig. 5:**
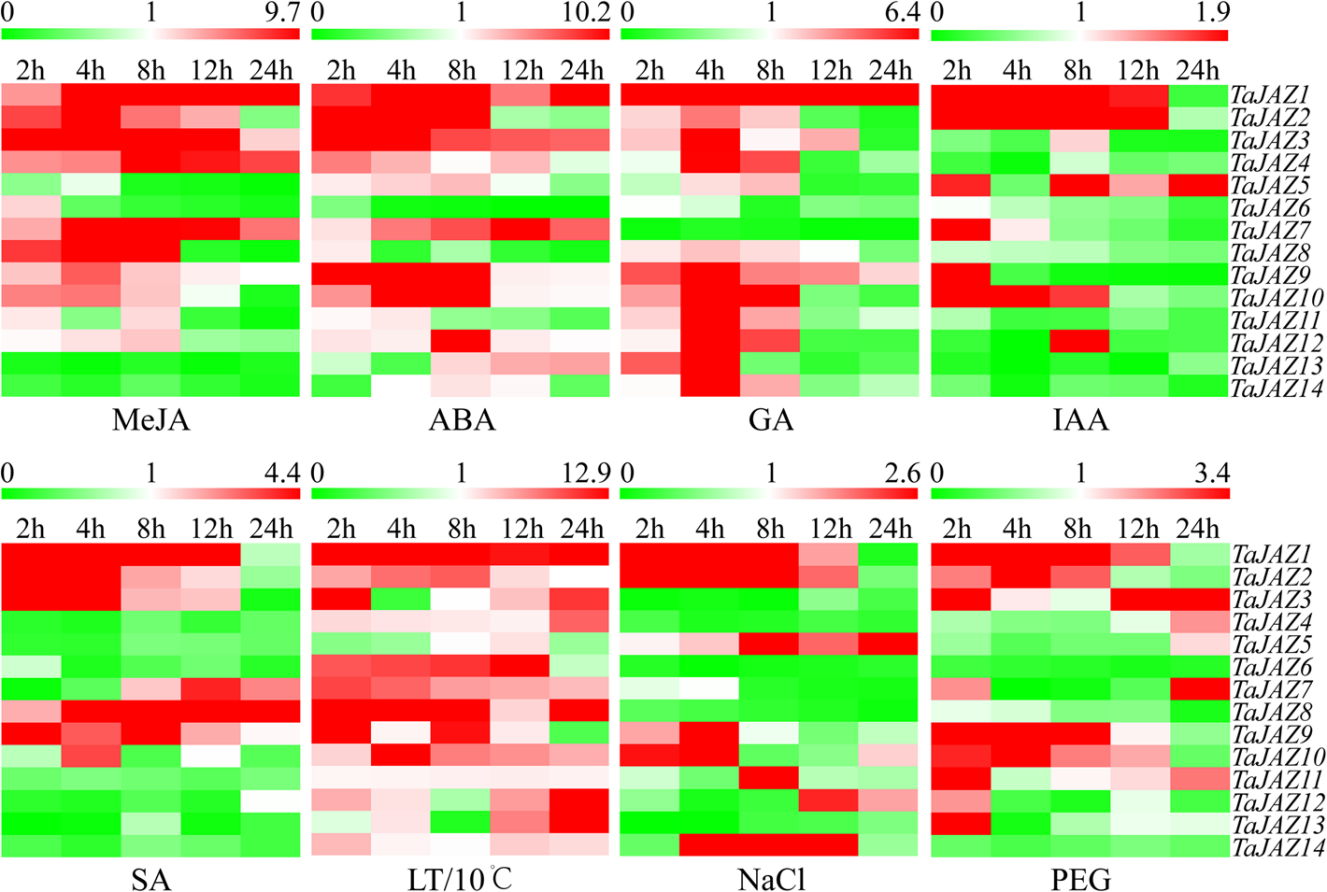
Expression heat maps of 14 *TaJAZ* genes under 5 phytohormone (MeJA, ABA, GA, IAA and SA), drought, salt and cold treatments in TGMS wheat line. qRT-PCR strategy was used to analyze the relative expression level of each *TaJAZ* gene. The expression level of wheat actin was used as the internal control to standardize the RNA samples for each reaction, and the expression at 0 h was set as 1 (data not show). The data are from three biological replicates

In order to obtain the expression profiling of *TaJAZ* genes under plant hormones, low temperature (10 °C), high salinity and drought treatments, qRT-PCR was performed to gain the relative expression pattern of each *TaJAZ* gene using the 14-day-old TGMS wheat seedlings. As shown in Fig. 5, the transcript profiling of *TaJAZ* genes showed up-regulation of *TaJAZ 1*, *2*, *3*, *4*, *7*, *8*, *9*, *10*, *11* and *12* and down-regulation for *TaJAZ5*, *6*, *13* and *14* in response to MeJA treatment. ABA treatment influenced the expression dynamics of *TaJAZ* genes. Except that *TaJAZ6*, *8* and *11* were inhibited, the expression levels of the remaining *TaJAZ* genes were up-regulated under ABA treatment (Fig. 5). Under GA treatment, the transcriptional levels of *TaJAZ1* and *9* were increased, and *TaJAZ6* and *7* were inhibited. The expression patterns of other *TaJAZ* genes were similar, i.e., up- at 2–4 h or 2–8 h followed by down-regulated at 24 h (Fig. 5). Under IAA treatment, the expression levels of *TaJAZ1*, *2* and *10* were up-regulated at time points 2 h, 4 h and 8 h; *TaJAZ7* and *9* were rapidly up-regulated at 2 h, but down-regulated subsequently; *TaJAZ5* was induced at each time point, but inhibited at 4 h. The expression levels of remaining *TaJAZ* genes were inhibited in different degree (Fig. 5). Moreover, the expression analysis of *TaJAZ* genes showed that *TaJAZ1*, *2*, *3*, *7*, *8* and *9* were up-regulated under SA treatment, and the rest of *TaJAZ* genes were down-regulated (Fig. 5). These revealed that all *TaJAZ* genes could respond to various plant hormones, and they might be involved in some complex signaling pathways.

Under high salinity treatment, the expression levels of *TaJAZ1*, *2*, *5* and *14* were up-regulated, while *TaJAZ9* and *10* were up-regulated at 2–4 h post treatment, but down-regulated at 8 h subsequently (Fig. 5). The expression levels of *TaJAZ12* were reduced at 2–8 h, and increased at 12–24 h. For drought stress, the expression analysis of *TaJAZ* genes showed that 6 genes (*TaJAZ1*, *2*, *3*, *9*, *10* and *11*) were up-regulated at 2–8 h or 2–12 h, whereas *TaJAZ6*, *8* and *14* were inhibited at each time point after treatment, and *TaJAZ4*, *5* and *7* were decreased firstly but increased subsequently (Fig. 5). In addition, except *TaJAZ5*, *11* and *14*, we found that *TaJAZ* genes could respond to low temperature (10 °C), and the transcriptional levels of the rest of genes were obviously up-regulated. Ulteriorly, we found that the expression levels of *TaJAZ1*, *2*, *4*, *7*, *8* and *10* were up-regulated at each time point (Fig. 5). These results indicated that the members of *TaJAZ* gene family were highly sensitive to high salinity, drought and low temperature treatments, and might be involved in some very complex regulation networks in TGMS wheat line BS366.

### Expression patterns of *TaJAZ* genes during the whole heading stage

**Fig. 6:**
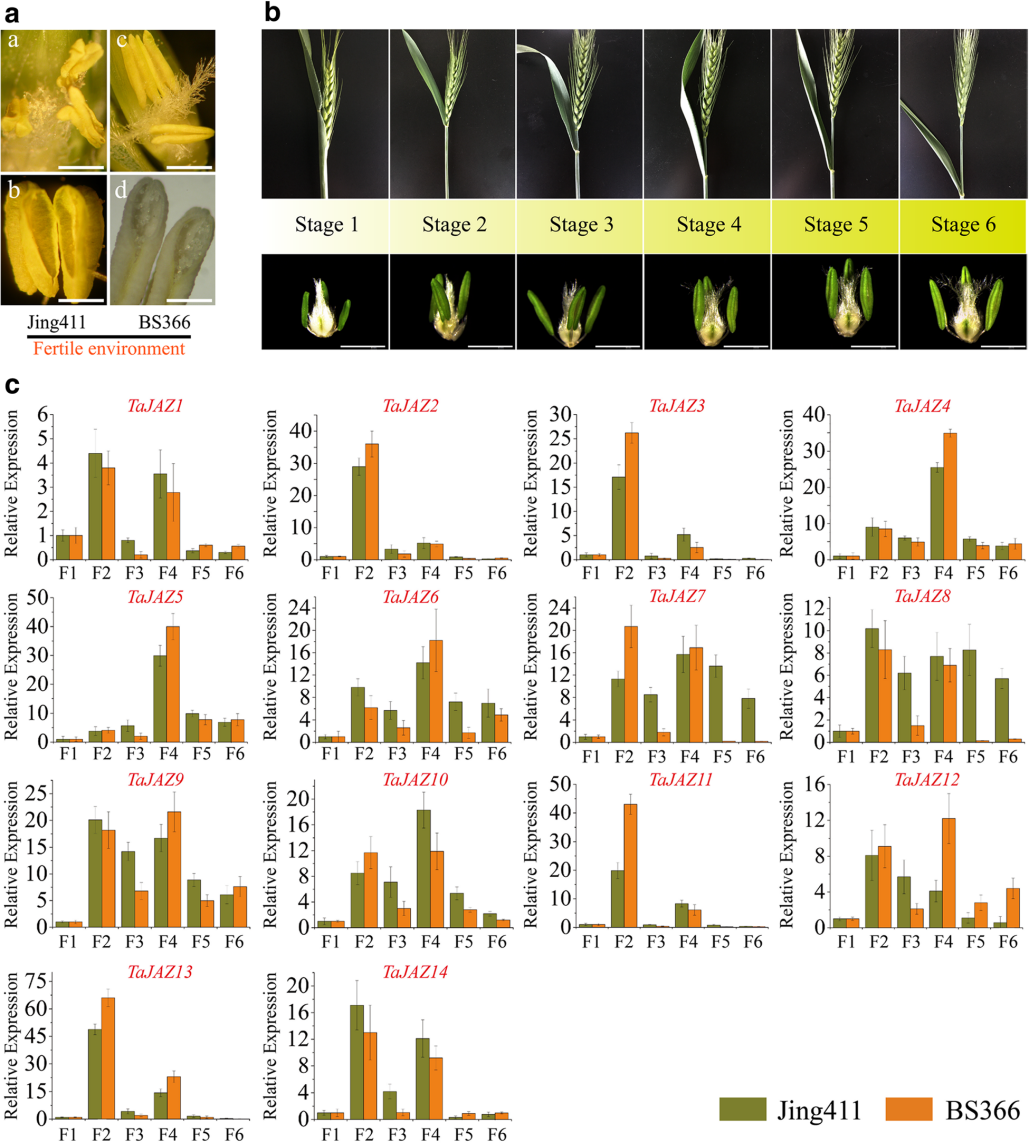
Expression patterns of *TaJAZ* genes during the whole heading stage. **A a**, **b** the phenotype of anther dehiscence in conventional wheat line Jing411; **c**, **d** the phenotype of anther dehiscence in TGMS wheat line BS366. Scale bars: a, c = 4 mm, b = 2 mm, d = 1 mm. **B** the schematic diagram of 6 stages during the whole heading period and the size of corresponding anther in each heading stage. Scale bars = 2 mm. **C** the relative expression patterns of *TaJAZ* genes in each heading stage. qRT-PCR was used to analyze the relative expression level of each *TaJAZ* gene. F1–F6 represent 6 stages during the whole heading period. The expression level of wheat actin was used as the internal control to standardize the RNA samples for each reaction, and the expression in stage 1 was set as 1. The data are from three biological replicates, and error bars represent the standard error

The TGMS wheat line BS366 exhibits temperature-dependent sterility. The anthers of BS366 are indehiscent in sterile environment (Fuyang, Anhui province, China) [48], and abnormal dehiscence in fertile environment (Beijing, China) (Fig. 6a, c and d). The anthers of the normal control wheat line Jing411 can normally dehisce in fertile environment (Fig. 6a, a and b). In order to explore the relationship between the expression patterns of *TaJAZ* genes and the abnormal anther dehiscence in TGMS wheat line BS366, the whole heading stage was divided into six periods, from the start of heading to pre-abloom stage (Fig. 6b). qRT-PCR was used to analyze the relative expression levels of each TaJAZ gene in BS366 and Jing411 anther tissues, which were collected from the fertile environment.

During the whole heading stage of BS366 and Jing411, the expression pattern of each *TaJAZ* gene was obviously fluctuant and regular (Fig. 6c). Except *TaJAZ5*, the relative expression levels of the rest of *TaJAZ* genes were markedly increased at the stage 2 and stage 4, and the corresponding expression patterns displayed some similarities (Fig. 6c). At the heading stage 6 of BS366, the transcriptional levels of 8 *TaJAZ* genes (*TaJAZ1*, *2*, *3*, *7*, *8*, *11 13* and *14*) were reduced compared with that in the heading stage 1, whereas the expression levels of *TaJAZ4*, *5*, *6*, *9*, *10* and *12* were increased (Fig. 6c). At the heading stage of Jing411, the expression levels of *TaJAZ1*, *2*, *3*, *11*, *12*, and *13* were inhibited, but the transcriptional levels of *TaJAZ4*, *5*, *6*, *7*, *8*, *9*, *10* and *14* were induced (Fig. 6c). Interestingly, the expression patterns of *TaJAZ7*, *8* and *12* were discrepant at the heading stage 6 of BS366 and Jing411, but similar at the heading stages 1–5 (Fig. 6c). These results revealed that the functions of *TaJAZ* genes were distinctly different, and TaJAZ*7*, *8* and *12* might play their conclusive roles in regulation of the degree of anther dehiscence at the heading stage 6 in wheat.

### Tissue-specific expression profiles of *TaJAZ* genes

**Fig. 7:**
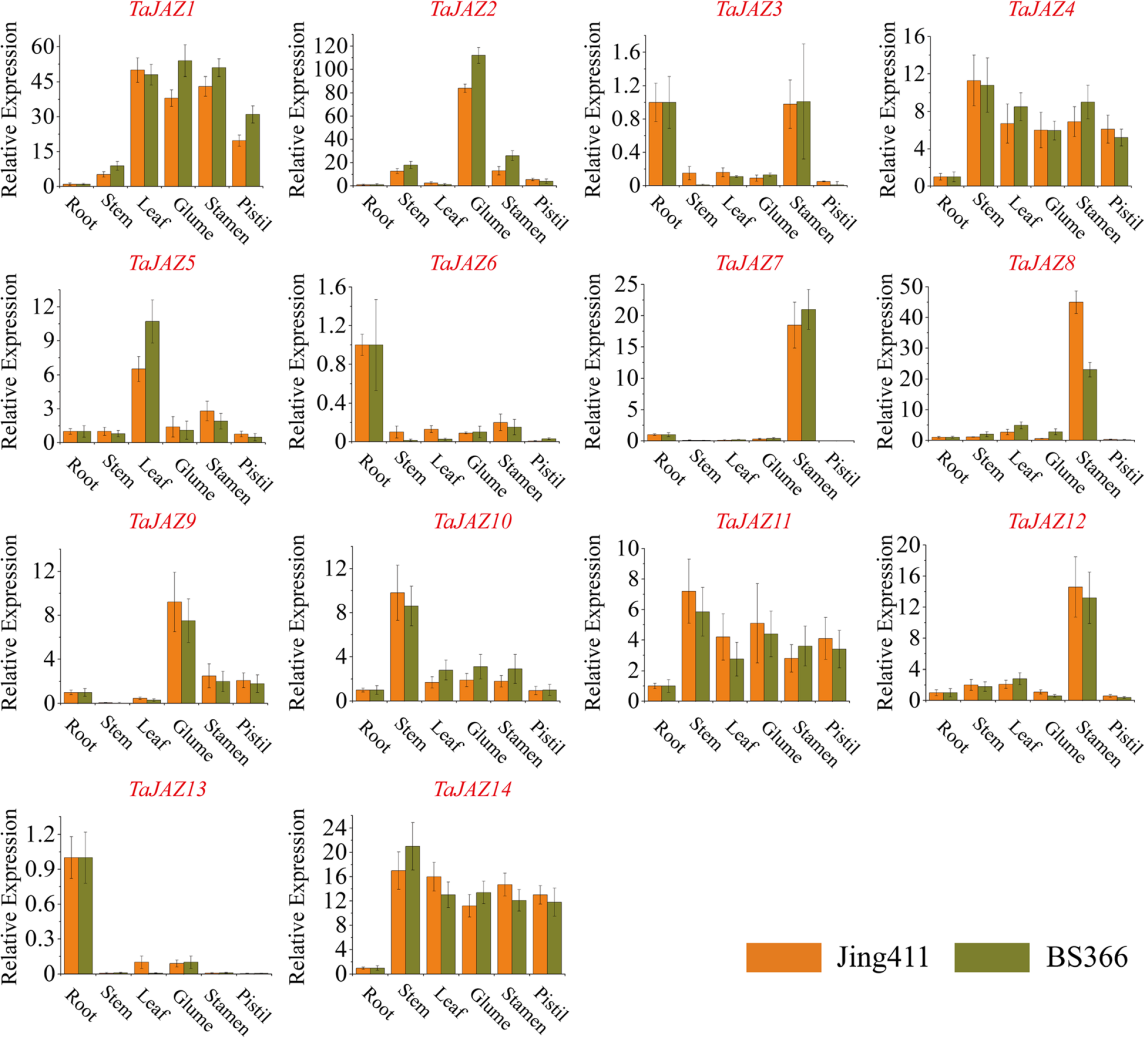
Real-time PCR analysis of *TaJAZ* genes in 6 wheat tissues in heading stage 6. The expression level of wheat actin was used as the internal control to standardize the RNA samples for each reaction, and the expression in the root was set as 1. The data are from three biological replicates, and error bars represent the standard error

The qRT-PCR was performed to investigate the tissue expression patterns of *TaJAZ* genes. The expression profiles of *TaJAZ* genes were analyzed in different tissues of the TGMS wheat line BS366 and the normal control Jing411 at heading stage 6 (Fig. 6b). As shown in Fig. 7, five *TaJAZ* genes (*TaJAZ1*, *4*, *10*, *11* and *14*) were expressed in root, stem, leave, stamen and pistil tissues, and they were expressed constitutively in BS366 and Jing411. Among the rest of *TaJAZ* genes, *TaJAZ6* and *13* were specifically expressed in root, *TaJAZ5*, was observably highly expressed in leaf tissues, and *TaJAZ2* and *9* were expressed specifically in glume (Fig. 7). The relative expression levels of *TaJAZ7*, *8* and *12* in stamen tissues were markedly higher than those in other tissues (Fig. 7). In addition, the expression level of *TaJAZ3* in root tissues was as high as that in stamen, and none of *TaJAZ* genes was specifically expressed in stem or pistil tissues (Fig. 7).

### Cloning of *TaJAZ7-D*, *8-D* and *12-D* and subcellular location

To investigate the reliability of identified *TaJAZ* genes, we used the specific primers to clone the ORF sequences of *TaJAZ7-D*, *8-D* and *12-D* and confirmed these sequences by DNA sequencing. The results showed that the ORF length and the nucleic acid constituent of *TaJAZ7-D*, *8-D* and *12-D* were in line with the data that identified from wheat genome database (Additional file 1: Figure S3A). Further, we confirmed the subcellular localization of TaJAZ7-D-GFP, TaJAZ8-D-GFP and TaJAZ12-D-GFP fusion proteins. As expected, all three fusion proteins localized in nucleus in Arabidopsis mesophyll protoplasts cells (Additional file 1: Figure S3B). These revealed that the *TaJAZ7-D*, *8-D* and *12-D* were true genes, and directly participated in JA signaling pathway in wheat.

## Discussion

### The structural characteristics of JAZ transcription repressors family in bread wheat

To date, the *JAZ* gene family has been reported in many plants, such as *A. thaliana* [24] and *H. brasiliensis* [49], *B. distachyon* [45] and *O. sativa* [43]. The expression patterns, gene and protein characteristics and the function of some *JAZ* genes have already been unfolded. However, the relative research on *JAZ* gene family was still infrequent in wheat. Based on the latest draft of wheat genome data, we isolated a *JAZ* gene family including 14 members from wheat (Table 1).

In plants, the gene construction of *JAZ* is manifold, and this multiformity is mainly reflected in the length and number of introns [50]. The length of introns in *TaJAZ* gene family ranged from 54 to 2098 bp. The shortest intron was only 54 bp in *TaJAZ7-B*, while the longest was 2098 bp in *TaJAZ14-D* (Fig. 3). Previous study revealed big gaps in the length of introns in *JAZ* gene family in many plants, ranging from 62 to 4422 bp [50]. We found that the intron length in *TaJAZ* gene family was in line with this range. In terms of the number of introns, the *TaJAZ* gene family members had 0–7 introns. *TaJAZ5* had the maximum 7 introns, whereas *TaJAZ6* and *13* had no introns (Fig. 3). This result was consistent with the number of introns in the *JAZ* genes of other plants [50]. However, some discrepancies were found in the number of introns among different copies of the same *TaJAZ* gene. For example, among the three copies of *TaJAZ4*, *TaJAZ4-A* and *-B* had 3 introns each, whereas *TaJAZ4-D* had 7 introns. *TaJAZ14-A* and *-D* had 6 introns each, whereas *TaJAZ14-B* had 4 introns. The similar characteristics were also found in the copies of *TaJAZ9* and *10* (Fig. 3). This characteristic of gene structure was proper in wheat, because wheat has sub-genomes A, B and D. Overall, the results mentioned above suggested that the phenomenon of intron indels or intron lose occurred in *TaJAZ* genes during the long evolutionary process, resulting in various structures of *TaJAZ* genes.

Generally, a typical JAZ protein has one TIFY and one Jas domain at its N- and C-terminal, respectively. TIFY domain has two main functions, with one as a medium for the interaction of homomeric and heteromeric complexes formation, and the other as a medium for the interaction between JAZ proteins and MYC transcription factors [24]. The other distinguishing feature of JAZ proteins is the highly conserved Jas domain located near the C-terminus [30]. It is well known that Jas domain participates in protein–protein interaction with both COI and transcription factors, such as MYC2 [26, 51], and the nuclear localization signal (NLS) is included in this domain sequence [52]. In the present study, all TaJAZ proteins contained one TIFY and one Jas domain, but the amino acid composition of TIFY and Jas domain was different. The multiple sequence alignment showed that some amino acid substitutions existed in the core sequence of TIFY domain (Additional file 1: Figure S1). For example, the protein sequence “TIFY” was replaced with “TVFY” in TaJAZ3, “TMFY” in TaJAZ8, and “TLFY” in TaJAZ4. This architectural feature is familiar in JAZ proteins in many other plants [43, 45, 49]. The protein sequences of Jas domain were relatively conserved, and the sequence lengths were almost coincident in Arabidopsis and gramineous rice and maize [24, 43, 44]. But in wheat, the Jas motif sequences of some *JAZ* proteins, such as TaJAZ4, 5 and 14, were inserted by a short and highly similar NLS-like peptide, respectively (Additional file 1: Figure S1). This structural feature was rare in other plant JAZ protein, and this specific structure was only available in wheat JAZ proteins.

In conclusion, there were some similarities and otherness in the characteristics between *TaJAZ* gene family and other plant *JAZ* gene families. The results mentioned above suggested that the *TaJAZ* gene family might have multiple functions due to the complex structural characteristics as same as *JAZ* gene families in many other plants.

### The putative functions of *TaJAZ* gene family

In order to gain more insights into the function of *TaJAZ* genes, we analyzed the *cis*-acting regulatory elements composition and the expression patterns under various stresses. *Cis*-acting regulatory elements are important molecular switches involved in the regulation of gene transcription under abiotic or biotic stresses [53]. In this study, we mainly detected the *cis*-acting regulatory elements that can respond to plant hormones (ABA, IAA, MeJA, GA and SA) and stress tolerances (low temperature, drought and high salinity). Due to the restriction of genomic sequencing data, we failed to gain the promoter region of *TaJAZ11*. In wheat *JAZ* gene promoter regions, the differences were mainly in the number and type of *cis*-acting regulatory elements. For example, the promoter region of *TaJAZ1* had 4 types of *cis*-acting regulatory elements, i.e. ABRE (the number was 4), TGA (the number was 2), box-w1 (the number was 2) and CGTCA-motif (the number was 1), while *TaJAZ6* only had 2 ABRE and 2 MBS elements (Additional file 2: Table S3). The promoter sequences of some *TaJAZ* genes lacked some types of *cis*-acting regulatory elements, but the expression patterns revealed that all *TaJAZ* genes could respond to all 8 treatments. For example, we found that all *TaJAZ* genes had no high salinity response-related *cis*-acting regulatory elements, but the relative expression levels of all *TaJAZ* genes were up- or down-regulated under high salinity treatment (Fig. 5). This indicated that the gene expression level under different treatments was not only dependent on the presence of relevant *cis*-acting regulatory elements, but also might be regulated by other physiological pathways in wheat.

To further investigate the biological functions of *TaJAZ* genes, we used different phytohormone to treat the wheat seedlings. Under MeJA treatment, the relative expression levels of *TaJAZ1*, *2*, *3*, *4*, *7*, *8*, *9*, *10* and *12* were obviously increased, while *TaJAZ5*, *6*, *11*, *13* and *14* were inhibited. By combining the *cis*-acting regulatory elements analyzed results, we found that the promoter sequences of *TaJAZ2*, *4* and *6* lacked the MeJA-responsive elements. Thus, we assumed that *TaJAZ1*, *3*, *5*, *7*, *8*, *9*, *10*, *12*, *13* and *14* were directly involved in JA signaling pathway in wheat, and *TaJAZ2*, *4* and *6* could also respond to MeJA treatment by other unknown regulative pathways. Likewise, for GA treatment, the relative expression levels of *TaJAZ6* and *7* were depressed, and the rest of *TaJAZ* genes were up-regulated. Based on the composition of *cis*-acting regulatory elements in promoter sequences, only *TaJAZ3*, *5*, *7* and *12* had GARE element involved in GA response, but the *TaJAZ* genes lacking GARE element could also respond to GA. For IAA treatment, only *TaJAZ5*, *7*, *8*, *12* and *13* had the TGA-element, but all *TaJAZ* genes could obviously respond to it. For SA treatment, only *TaJAZ3*, *7*, *8*, *10* and *13* had the TCA-element, but the response was available in all genes. For ABA treatment only *TaJAZ2*, *4*, *6*, *8* and *12* had no ABRE element, but they were still sensitive to the presence of ABA (Fig. 5 and Additional file 2: Table S3). Given this, we found that some *TaJAZ* genes could respond to various phytohormones, and they might be directly or indirectly involved in the phytohormone crosstalk.

It is clear that the phytohormone crosstalk is universally available in plants, and a very complex signaling regulative network, which constituted by many phytohormone crosstalk, plays a very important role in the regulation of growth and development in plants. There is no doubt that JA signaling pathway plays a leading role in connecting different phytohormone signaling pathways [33]. As a repressor in downstream of JA signaling, JAZ proteins were irreplaceable, and they are the linkers among different crosstalks [33]. By synthesizing the analytic results of the *cis*-acting regulatory elements composition and the expression patterns under abiotic stresses (Fig. 5 and Additional file 2: Table S3), we speculated that *TaJAZ3*, *5*, *7* and *12* were involved in the JA-GA crosstalk, which is a crosstalk to promote plant growth and defense against pathogens. Moreover, the JA-GA crosstalk can also act synergistically during stamen development, and JAZ proteins appear to play a significant role in this developmental function by interacting with the transcription factors *MYB21* and *MYB24*, which both required for JA- and GA-mediated stamen development and male fertility [54]. Therefore, we thought that *TaJAZ3*, *5*, *7* and *12* had a close contact with the development of stamen in wheat. The mechanism of JA-SA crosstalk in plant remains largely unknown, but the previous evidence has shown the effect of SA on JA signaling through direct or indirect regulation of the stabilization of JAZ proteins [55, 56]. This crosstalk can protect plants from the biotic and abiotic stresses, especially the pathogen infection [57, 58]. Here, we found that *TaJAZ3*, *7*, *8*, *10* and *13* had two types of *cis*-acting regulatory elements CGTCA-motif and TCA-element, and they were sensitive to JA and SA treatments (Fig. 5 and Additional file 2: Table S3). Thus, we thought that the functions of *TaJAZ3*, *7*, *8*, *10* and *13* were to enhance or inhibit the antiviral ability of plants. IAA is a very important phytohormone and plays vital roles during the development of plants [59]. The evidences revealed that the function of JA-IAA crosstalk was mainly embodied in regulating the root meristem activity and stem cell maintenance via antagonistic effect in plants [60–62]. Based on the results of *cis*-acting regulatory element component and the expression patterns (Fig. 5 and Additional file 2: Table S3), we assumed that *TaJAZ5*, *7*, *8*, *12* and *13* were directly participated in the JA-IAA crosstalk, and they might be involved in the regulation of primary root growth in wheat. ABA is a “stress hormone” that can regulate growth, stress tolerance, seed germination and senescence in plants. In JA-ABA crosstalk, both synergetic and antagonistic interactions are well known, and it is possible that JAZ proteins play important roles in regulating the JA-ABA crosstalk [33]. Given this, except *TaJAZ2*, *4*, *6*, *8* and *12*, which lacked ABRE element, we speculated the rest of *TaJAZ* genes took part in the JA-ABA crosstalk, and played a role in plant stress tolerance, such as high salinity, drought and low temperature stresses (Fig. 5 and Additional file 2: Table S3).

In addition, the analysis of phylogenetic relationship among the *JAZ* genes can also reveal the putative function of them, because the homologous genes usually have similar biological functions [63]. In this study, we found that TaJAZ2 was clustered with OsJAZ1 into the sub-group G9 (Fig. 4). It is clear that OsJAZ1 protein interacts with a basic helix-loop-helix protein, OsbHLH148, to regulate the drought tolerance in rice [64]. Here, we found 4 drought-inducible *cis*-acting regulatory elements MBS in the promoter sequence of *TaJAZ2*, and the expression level of *TaJAZ2* was also variational under drought stress. Thus, *TaJAZ2* was likely participated in drought tolerance in wheat. Then, we found that TaJAZ4 was clustered with BdJAZ5 into the sub-group G3 (Fig. 4). The expression levels of *BdJAZ5* are increased under salt, cold and heat treatments in *B. distachyon* [45]. There were 2 low temperature related *cis*-acting regulatory elements LTR in the promoter region of *TaJAZ4*, and the expression pattern of *TaJAZ4* was similar with that of *BdJAZ5* under cold stress, so *TaJAZ4* might be involved in cold response in wheat. Further, we found that TaJAZ6 shared a high similarity with BdJAZ7 in sub-group G10 (Fig. 4), and there were 2 MBS elements in its promoter sequence. The expression patterns of *TaJAZ6* and *BdJAZ7* were similar under drought stress (Fig. 5) [45], indicating that *TaJAZ6* was also involved in drought tolerance in wheat. Overall, the functions of many *TaJAZ* genes were overlapping, which needed to be studied minutely in the future.

### The evolution analysis of JAZ transcription repressors family

In the ancient terricolous plants, there are 7 and 6 members in the *JAZ* gene family in *P. patens* and *S. moellendorffii* genome, respectively [50]. In neonatal terricolous plants, there are 13 *JAZ* genes in gymnospermous *P. stichensis* genome, 12 *JAZ* genes in dicotyledonous *A. thaliana* genome, 15, 16 and 15 *JAZ* genes in gramineous *B. distachyon*, *S. bicolor*, and *O. sativa* genomes, respectively [50]. In the present study, 14 *TaJAZ* genes were identified from wheat genome (Table 1), and this number was similar to those in gramineous plants, but obviously more than those in older plants *P. patens* and *S. moellendorffii*. This result suggested that the number of *JAZ* genes in higher plants undergone the expansion, and became stable subsequently. Based on the analysis of chromosome localization, we found that the chromosome distribution of *TaJAZ* genes was tufted. For example, the copies of *TaJAZ4*, *5*, *6* and *7* were densely distributed on the chromosomes 4A, 4B and 4D, respectively (Fig. 2). In addition, both TaJAZ4 and 5 were clustered into sub-group G3 (Fig. 4), while TaJAZ6 and 7 were clustered into sub-groups G10 and G6 (Fig. 4), respectively, indicating that TaJAZ4 and 5 undergone the event of tandem duplication, and maybe the event of divergence happened in the evolutionary process of *TaJAZ* genes. Moreover, we found that a pair of tandem *TaJAZ* genes, *TaJAZ13*-*D1* and -*D2*, shared a high similarity in their protein sequences (Additional file 1: Figure S1), exhibiting that the duplication event of these two genes also happened. Given this, we thought that the gene tandem duplication was the main result leading to the augmentation in the number of *TaJAZ* gene.

Based on the N-J phylogenetic tree, all JAZ proteins from different plants were clustered into 11 subgroups (Fig. 4). The sub-groups G1, G2, G3, G5, G8 and G9 each had one SmJAZ protein, while none was present in the rest of sub-groups. Thus, we separated these 11 sub-groups into groups I (marked by red) and II (marked by blue) (Fig. 4). In group I, the sub-group G1 had only one JAZ protein, SmJAZ6, indicating that all JAZ proteins in terrestrial plants originated from a common ancestor. In sub-groups G3 and G5, the JAZ proteins from *S.moellendorffii* and other plants, such as *T. aestivum*, *B. distachyon* and *A. thaliana*, were clustered in these two clades, indicating that the differentiation of these JAZ proteins might have predated the divergence between flowering plants and pteridophyte (Fig. 4). In sub-groups G8 and G9, the JAZ proteins from *P. patens* and other plants, such as *A. thaliana*, and *O. sativa*, were clustered in these two sub-groups, suggesting that the differentiation of those JAZ proteins might have predated the divergence between bryophyte and tracheophyte (Fig. 4). In group II, the JAZ proteins from gymnospermous *P. sitchensis* and other angiosperm were included in G7, suggesting that those JAZ proteins might have predated the divergence between gymnosperms and angiosperms (Fig. 4). In addition, sub-groups G4, G6 and G10 comprised some JAZ proteins from monogenus and dicotyledonous plants, indicating that these JAZ proteins appeared before the divergence between monogenus and dicotyledonous plants (Fig. 4). The sub-group G11 only included the JAZ proteins from gramineous plants (Fig. 4), suggesting that the differentiation of these JAZ proteins ahead of the formation of gramineous plants.

For TaJAZ protein family, we found that all TaJAZ proteins were directly clustered with the JAZ proteins from *Ae. tauschii*, *B. distachyon*, *S. bicolor* or *O. sativa*. For example, TaJAZ3 was clustered with BdJAZ10 into sub-group G4; TaJAZ9 was clustered with AetJAZ2 into G6; TaJAZ10 was clustered with BdJAZ14 and SbJAZ11 into G7; and TaJAZ13 was clustered with BdJAZ6 and AetJAZ4, 5, 6, 7 and 8 (Fig. 4). This could be attributed to the fact that these 5 plant species are gramineous. Thus, we speculated that the differentiation of TaJAZ protein family occurred after the divergence between monocotyledon and dicotyledon (90 MYA), and ahead of the formation of gramineous plants (50-80 MYA). In addition, the Ka/Ks ratio revealed that the TaJAZ protein family undergone a process of purifying selection (Table 2), suggesting that the TaJAZ protein family tended to be stable during the long evolutionary process.

### *TaJAZ7*, *8* and *12* were involved in the abnormal anther dehiscence

It is clear that JA, as a kind of important phytohormone, is widely involved in the regulation of anther dehiscence, filaments elongation and pollen fertility in plants [65]. The JA biosynthesis and signaling pathways were important for the development of anther during late developmental stage [66]. JAZ proteins, as repressors in JA signaling pathway, inhibit the transcription of JA response genes [33], and there is no doubt that *JAZ* genes play an essential role in the JA-mediate regulation pathway of anther dehiscence in plants [66]. The TGMS wheat line BS366 is a temperature dependent variety and its fertility can convert under different environments [48]. Hybrid seed could be produced under sterile condition (TGMS line BS366 as maternal plant), while TGMS line itself could be propagated under fertile condition [41]. The anther dehiscence of conventional wheat line Jing411 is normal under fertile condition (Fig. 6a, a and b), and the pollen can spill out from anthers smoothly. In sterile environment, the anther of TGMS wheat line BS366 is absolutely indehiscent [48], resulting in the male sterility. Interestingly, we found that the anther of BS366 could not fully dehisce, and only the topmost part of anther could crack (Fig. 6a, c and d), leading to the pollen spilling out incompletely. This abnormal phenotype was profitless for the seed multiplication of TGMS wheat line BS366.

In order to explore the potential relationship between the expression patterns of *TaJAZ* genes and the phenomenon of abnormal anther dehiscence, we divided the heading stage into six periods, and the expression pattern of each *TaJAZ* gene was checked in the anther tissues of BS366 and Jing411.. As shown in Fig. 6c, the expression patterns of all *TaJAZ* genes were highly similar from stage 1 to stage 5, and the relative expression levels in stage 2 and stage 4 increased obviously. Given this, we speculated that all 14 *TaJAZ* genes played important roles in regulation of anther development at stages 1, 2, 3, 4 and 5. Further, we noticed that the relative expression levels of *TaJAZ* genes at stage 6 were distinguishing in the anther of BS366, and the expression patterns were mainly divided into two types. The first category included *TaJAZ1*, *2*, *3*, *7*, *8*, *11*, *13* and *14*, and the expression levels of these genes were inhibited (Fig. 6c). The second category included *TaJAZ4*, *5*, *6*, *9*, *10* and *12*, and the expression levels of these genes were induced (Fig. 6c). In the anther of Jing411, the expression patterns of *TaJAZ1*, *2*, *3*, *4*, *5*, *6*, *9*, *10*, *11*, *13* and *14* were as same as those in BS366 at the heading stage 6 (Fig. 6c). It was obvious that the expression levels of *TaJAZ7*, *8* and *12* in the anther of Jing411 were adverse to those in BS366 at stage 6. For example, in the anther of BS366, the expression level of *TaJAZ7* was inhibited at stage 6, exhibiting 18 times lower than that at stage 1 (Fig. 6c). In contrast, the transcriptional level of *TaJAZ7* increased at the heading stage 6 in the anther of Jing411, displaying 7 to 8 folds higher than that at stage 1 (Fig. 6c). Similar results were also found in the expression patterns of *TaJAZ8* and *12*. These indicated that not all *TaJAZ* genes were involved in the regulation of anther dehiscence, and the genes with the same expression patterns in the anthers of Jing411 and BS366 may not be involved in the regulation of the anther dehiscence.

Moreover, the tissue-specific expression assay was performed to check the expression levels of *TaJAZ* genes in different tissues of BS366 and Jing411.. The relative expression levels of *TaJAZ1*, *2*, *4*, *10*, *11* and *14* were obviously high in stamen tissues, but they all had high expression levels in other tissues (Fig. 7). Therefore, we thought that the regulatory effect of these genes was constitutive. The relative expression levels of *TaJAZ7*, *8* and *12* were markedly higher in stamen than that in other tissues, showing a high degree of expression specificity. For most of *TaJAZ* genes, the tissue-specific expression patterns were consistent in the anther tissues of Jing411 and BS366. In addition, the subcellular localization showed that *TaJAZ7, 8* and *12* were all located in nucleus (Additional file 1: Figure S3). Based on the results mentioned above, we thought that *TaJAZ7*, *8* and *12* were directly participated in JA signaling pathway, and most likely to directly regulate the abnormal anther dehiscence. Thus, *TaJAZ7*, *8* and *12* were regarded as the candidate genes for the regulation of abnormal anther dehiscence in TGMS wheat line. The functions of *TaJAZ7*, *8* and *12* will be analyzed in our future works.

## Conclusions

Fourteen *JAZ* family genes were identified from common wheat genome. The structure analysis revealed that *TaJAZ* gene family had unique characteristic in protein structure. JAZ proteins from wheat and some other plants were classified into 11 different orthologous groups, showing that all JAZ proteins from terrestrial plants derived from the same ancestor, and TaJAZ proteins shared high similarity with the JAZ proteins from *Ae. tauschii*, *B. distachyon*, and *O. sativa*. The Ka/Ks ratio of *TaJAZ* family genes were very low (less than 1), suggesting that these genes had been under purifying selection in the evolutionary histories.

Moreover, we found that wheat *JAZ* genes showed differential tissue-specific expression patterns responsive to abiotic stresses. *TaJAZ7*, *8* and *12* directly participated in JA signaling pathway, and closely involved in the regulation of the abnormal anther dehiscence in TGMS wheat line. In conclusion, these results enriched our knowledge of *JAZ* gene family in plants, and provided novel candidate genes for improving the TGMS wheat line in seed reproduction.

## Methods

### The sources of sequence data

The whole-genome sequences of *Triticum aestivum* (*T. aestivum*, Ta) was downloaded from the wheat genome URGI database (http://wheat-urgi.versailles.inra.frl) and Ensembl database (http://plants.ensembl.org). The JAZ protein sequences of *Physcomitrella patens* (*P. patens*, Pp), *Selaginella moellendorffii* (*S. moellendorffii*, Sm), *Sorghum bicolor* (*S. bicolor*, Sb), *Brachypodium distachyon* (*B. distachyon*, Bd), and *Zea mays* (*Z. mays*, Zm) were obtained from JGI database (http://genome.jgipsf.org/). The JAZ protein sequences of *Picea sitchensis* (*P. sithensis*, Ps) were achieved from NCBI database (http://www.ncbi.nlm.nih.gov/). The JAZ protein sequences of *Arabidopsis thaliana* (*A. thaliana*, At) were acquired from TIAR database (http://www.arabidopsis.org/). The JAZ protein sequences of *Oryza sativa* (*O. sativa*, Os) were collected from TIGR database (http://www.tigr.org/tdb/ezkl/). The JAZ protein sequences of *Hevea brasiliensis* (*H. brasiliensis*, Hb) were isolated following Hong et al. [49]. The JAZ protein sequences of *Aegliops tauschii* (*Ae. tauschii*, Aet) were isolated according to our previous study [67]. All information of sequences was listed in Additional file 2: Table S2.

### Identification of *JAZ* gene family members in wheat

Based on the wheat genome sequences, a local nucleotide and protein database was established by NCBI local BLAST program (Ftp://ftp.ncbi.nlm.nih.gov/blast/executables/blast+/LATEST/). The hidden Markov model (HMM) profiles PF06200 (TIFY domain) and PF09425 (Jas domain) of the *JAZ* family were extracted from the Pfam database (http://pfam.sanger.ac.uk) and these two HMM profiles were used to search the local wheat protein database for target hits with the TIFY domain and Jas domain by HMMER 3.0 (http://hmmer.janelia.org/). All non-redundant sequences with E-values lower than 1.0E-05 were selected and received a conserved domain check using the SMART web server (http://smart.emblheidelberg.del) and Pfam tool (http://pfam.xfam.org/). Then, the coding sequences and genome sequences of *TaJAZ* genes were extracted from the local nucleotide database using the sequence ID of TaJAZ proteins.

### Analysis of gene structures, protein motifs and *cis*-acting regulatory elements

To illustrate the structures of *TaJAZ* genes, the coding sequence of each *JAZ* gene was aligned with its genomic sequence using the Gene Structure Display Server (GSDS) program (http://gsds.cbi.pku.cn/index-php). Multiple Exprectation Maximization for Motif Elicitation (MEME) was used to identify the motifs of TaJAZ proteins with default settings. To analyze the putative *cis*-acting elements in a promoter region, 1.5 kb region upstream of the start codon in each *TaJAZ* gene was scanned in the PlantCARE database (http://bioinformatics.psb.ugent.be/webtools/plantcare/html/).

### Phylogenetic relationship, chromosome distribution and naming convention of *TaJAZ* genes

In order to understand the phylogenetic relationship of *TaJAZ* genes, the unrooted phylogenetic tree was built using MEGA 6.0 via the Neighbor Joining (NJ) method. The position of each *JAZ* gene in the corresponding chromosome was confirmed by BLAST, and the alignment results were displayed using the MapInspect software (http://mapinspect.software.informer.com/). Duplicated genes in the branch ends of each group belonging to the A, B or D sub-genomes of wheat were considered as the homologous copies of the same *JAZ* gene. All *TaJAZ* genes were named according to their chromosome position and homology among the three wheat sub-genomes [68].

### Multiple sequence alignment and phylogenetic analysis of TaJAZ proteins

Multiple sequence alignment of the candidate TaJAZ proteins was performed using the Clustal X software (Version 1.81) with default settings. Meanwhile, 6 SmJAZ, 12 AtJAZ, 15 OsJAZ, 7 PpJAZ, 6 ZmJAZ, 15 BdJAZ, 15 SbJAZ, 13 PsJAZ, 8 AetJAZ and 7 HbJAZ proteins were included in the phylogenetic analyses. The unrooted tree was performed using the NJ method of MEGA 6.0 software with bootstrap values from 1000 replicates.

### Ka and Ks calculations

The Ka and Ks calculation method was described previously [68]. Briefly, the orthologous *JAZ* gene pairs between wheat and *B. distachyon* were used to calculate Ka and Ks in the PAL2NAL server using the codeml program of phylogenetic analysis by maximum likelihood [69]. The *B. distachyon* was used as an outgroup.

### Plant materials, treatments and sample collections

The thermo-sensitive genic male sterile (TGMS) wheat line BS366 and normal wheat line Jing411 were planted in the experimental fields in Beijing (China, N 39°54′, E 116°18′), and managed conventionally. For expression pattern analysis of *TaJAZ* genes in Jing411 and BS366, the anther tissues were sampled at six stages during the whole heading periods (Fig. 6b).

For tissue-specific expression analyses, 6 tissues (root, stem, leaf, glume, stamen and pistil) from Jing411 and BS366 were collected at the heading stage 6 (Fig. 6b). For abiotic stresses, BS366 seeds were germinated at 25 °C, then the seedlings were cultured in green house for 16 h light/8 h dark at 25 °C. After 2 weeks, the seedlings were sprayed with 2 mM SA, 100 mM MeJA, 100 mM GA3, 50 mM IAA, and 100 mM ABA that were dissolved in 0.1% (*v/v*) ethanol. The control plants were treated with 0.1% (*v/v*) ethanol. The leaf tissues from seedlings were collected at 0, 2, 4, 8, 12 and 24 h post treatment. For the high-salinity and drought treatments, the roots of wheat seedlings were soaked in 200 mM NaCl and PEG6000 (-0.5 MPa), respectively. The leaf tissues from seedlings were sampled at 0, 2, 4, 8, 12 and 24 h post treatment. For low temperature stress, the 2-week-old seedlings were moved to the incubator in which the temperature was set at 10 °C. The leaf tissues were collected at 0, 2, 4, 8, 12 and 24 h post treatment. All the samples mentioned above were rapidly frozen in liquid nitrogen and stored at -80 °C freezer for RNA extraction.

For subcellular localization, Arabidopsis ecotype Col-0 was used for protoplast preparation. Arabidopsis seedlings were planted in greenhouse with a 16 h light/8 h dark and a 22/20 °C day/night temperature cycle.

### Total RNA extraction and qRT-PCR

For the expression analysis of *TaJAZ* genes, total RNA was isolated from wheat tissues using TRIzol reagent (Invitrogen, USA) according to the manufacturer’s instructions. First-strand cDNA synthesis was performed using a PrimeScript^TM^ RT Reagent Kit with gDNA Eraser (TaKaRa, Japan). qRT-PCR was carried out using an ECO Real-time PCR system (Illumina, USA) with SYBR® Permix Ex Taq^TM^ (TaKaRa, Japan). Primer premier 5.0 program was used to design the primers. Wheat Actin gene (GenBank accession: AB181991) was used as the reference control.

The qRT-PCR for each assay was set up as a 10-μL reaction mixture containing 1.0 μL of cDNA, 5 μL of fluorescent reagent SYBR, 3 μL of ddH_2_O, and 0.5 μL each of forward and reverse primers. The reactions were as follows: 95 °C for 30 s, 45 cycles of 95 °C for 5 s and 58 °C for 30 s. For the melting curve analysis, a program of 95 °C for 15 s followed by a constant increase from 55 °C to 95 °C was included after the PCR cycles. The expression analysis of *TaJAZ* genes and reference gene was performed using the same PCR program as detailed above or with a slightly adjusted annealing temperature. The relative expression levels of *TaJAZ* genes were detected using the comparative threshold cycle method 2 ^–ΔΔCT^ [70]. The primers are listed in Additional file 2: Table S1, and the analysis was confirmed in triplicate.

### Cloning of *TaJAZ7-D*, *TaJAZ8-D* and *TaJAZ12-D*, subcellular localization and morphological observation

The nucleotide sequences of *TaJAZ7-D*, *8-D* and *12-D*, retrieved from local wheat nucleotide database, were used for designing specific primers to amplify the corresponding ORF sequences. Then, the ORF sequences of the three genes were inserted into pMD18-T vector, and confirmed by DNA sequencing. Next, the three ORF sequences (no termination codon) with restriction enzyme sites were amplified by PCR strategy and the products were digested using corresponding enzymes. Finally, the ORF sequences of the three *TaJAZ* genes were inserted into the 16318hGFP (p35S::GFP) vector and produced the vectors p16318-*TaJAZ7-D*, p16318-*TaJAZ8-D* and p16318-*TaJAZ12-D*, respectively. Arabidopsis mesophyll protoplasts cells were isolated according to Yoo et al. [71]. The three fusion plasmids or control vector 16318hGFP were transformed into Arabidopsis mesophyll protoplasts cells using the PEG4000-mediated method [72]. GFP signal was detected by laser confocal fluorescence microscopy (ZEISS LSM 880, Germany). The representative spikelets and anthers were imaged using a Leica MZ16F stereomicroscope (Leica Microsystems, Wetzlar, Germany). The spikes of wheat seedlings were imaged using a digital SLR camera (Canon, EOS 700D).

## Additional files

Additional file 1:
**Figure S1.** The multiple sequence alignment of TaJAZ proteins. The pink and green lines represent the core sequence of TIFY domain and Jas domain in each of TaJAZ protein. The blank boxes represent the NLS sequence in each of TaJAZ protein. The location of TIFY domain and Jas domain were also marked out in each of TaJAZ protein. **Figure S2.** Phylogenetic relationship of JAZ between *B. distachyond* and wheat. The BdJAZs in the indicated groups were used as an outgroup to calculate Ka and Ks. **Figure S3.** The PCR amplified products and subcellular location of *TaJAZ7-D*, *TaJAZ8-D* and *TaJAZ12-D*. (A) lane 1, 3 and 5 represent negative control;. lane 2: the PCR amplified product of *TaJAZ7-D*; lane 4: the PCR amplified product of *TaJAZ8-D*; lane 6: the PCR amplified product of *TaJAZ12-D*. (B) the subcellular locations of TaJAZ7-D-GFP, TaJAZ8-D-GFP and TaJAZ12-D-GFP fusion proteins. Scale bars = 20 μm. (ZIP 3354 kb)
Additional file 2:
**Table S1.** The primers used in this study. **Table S2.** The information of *JAZ* or *JAZ*-like genes in different plants. **Table S3.** The number and composition of *cis*- acting regulatory elementsof each *TaJAZ* gene. (ZIP 40 kb)

## Abbreviations

ABA: Abscisic acid
GA: Gibberellin
GFP: Green fluorescent protein
IAA: Indole-3-acetic acid
JAZ: Jasmonate-ZIM domain
MeJA: Methyl jasmonate
MYA: Million years ago
ORF: Open reading frame
qRT-PCR: Quantitative real-time PCR
SA: Salicylic acid
SLR: Single lens reflex
TGMS: Thermosensitive genic male sterile
