# Supplementary figures and images for: Genome-wide characterization of JASMONATE-ZIM DOMAIN transcription repressors in wheat (Triticum aestivum L.)
Source: BMC Genomics. 2017 Feb 13;18:152. doi: 10.1186/s12864-017-3582-0 (PMC5307646; doi:10.1186/s12864-017-3582-0)

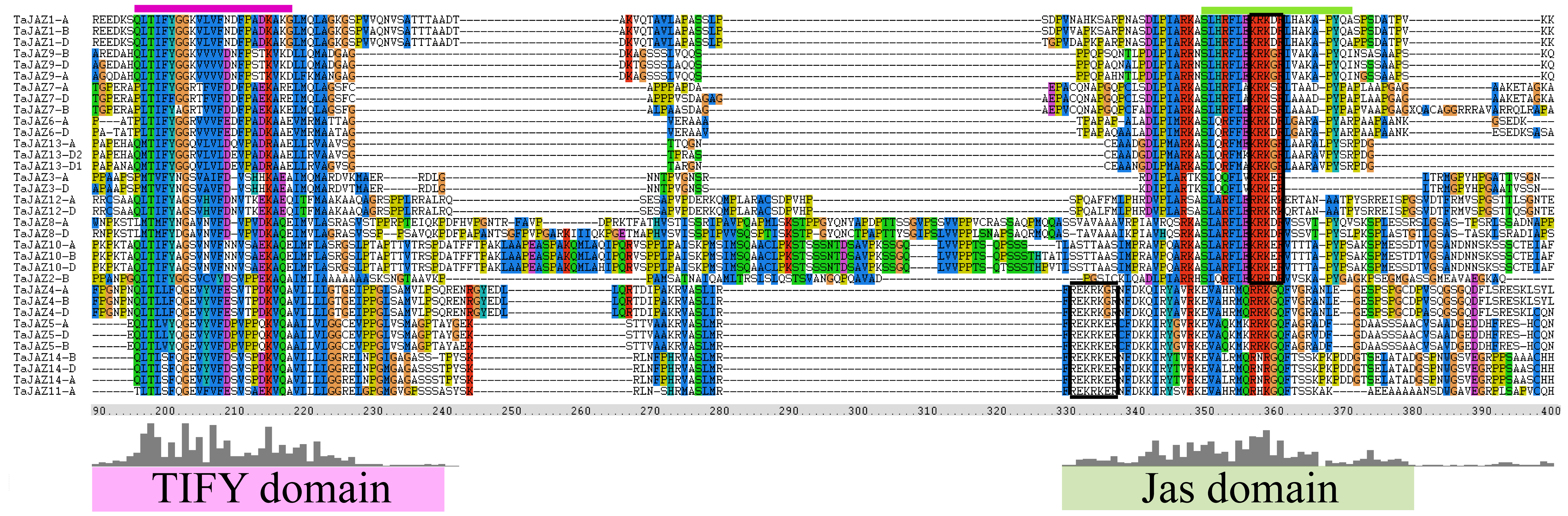

Supplement: Additional file 1: — Figure S1. The multiple sequence alignment of TaJAZ proteins. The pink and green lines represent the core sequence of TIFY domain and Jas domain in each of TaJAZ protein. The blank boxes represent the NLS sequence in each of TaJAZ protein. The location of TIFY domain and Jas domain were also marked out in each of TaJAZ protein. Figure S2. Phylogenetic relationship of JAZ between B. distachyond and wheat. The BdJAZs in the indicated groups were used as an outgroup to calculate Ka and Ks. Figure S3. The PCR amplified products and subcellular location of TaJAZ7-D, TaJAZ8-D and TaJAZ12-D. (A) lane 1, 3 and 5 represent negative control;. lane 2: the PCR amplified product of TaJAZ7-D; lane 4: the PCR amplified product of TaJAZ8-D; lane 6: the PCR amplified product of TaJAZ12-D. (B) the subcellular locations of TaJAZ7-D-GFP, TaJAZ8-D-GFP and TaJAZ12-D-GFP fusion proteins. Scale bars = 20 μm. (ZIP 3354 kb) [file 12864_2017_3582_MOESM1_ESM.zip › Figure S1.tif]

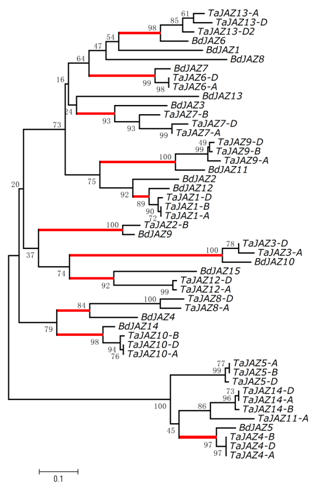

Supplement: Additional file 1: — Figure S1. The multiple sequence alignment of TaJAZ proteins. The pink and green lines represent the core sequence of TIFY domain and Jas domain in each of TaJAZ protein. The blank boxes represent the NLS sequence in each of TaJAZ protein. The location of TIFY domain and Jas domain were also marked out in each of TaJAZ protein. Figure S2. Phylogenetic relationship of JAZ between B. distachyond and wheat. The BdJAZs in the indicated groups were used as an outgroup to calculate Ka and Ks. Figure S3. The PCR amplified products and subcellular location of TaJAZ7-D, TaJAZ8-D and TaJAZ12-D. (A) lane 1, 3 and 5 represent negative control;. lane 2: the PCR amplified product of TaJAZ7-D; lane 4: the PCR amplified product of TaJAZ8-D; lane 6: the PCR amplified product of TaJAZ12-D. (B) the subcellular locations of TaJAZ7-D-GFP, TaJAZ8-D-GFP and TaJAZ12-D-GFP fusion proteins. Scale bars = 20 μm. (ZIP 3354 kb) [file 12864_2017_3582_MOESM1_ESM.zip › Figure S2.tif]

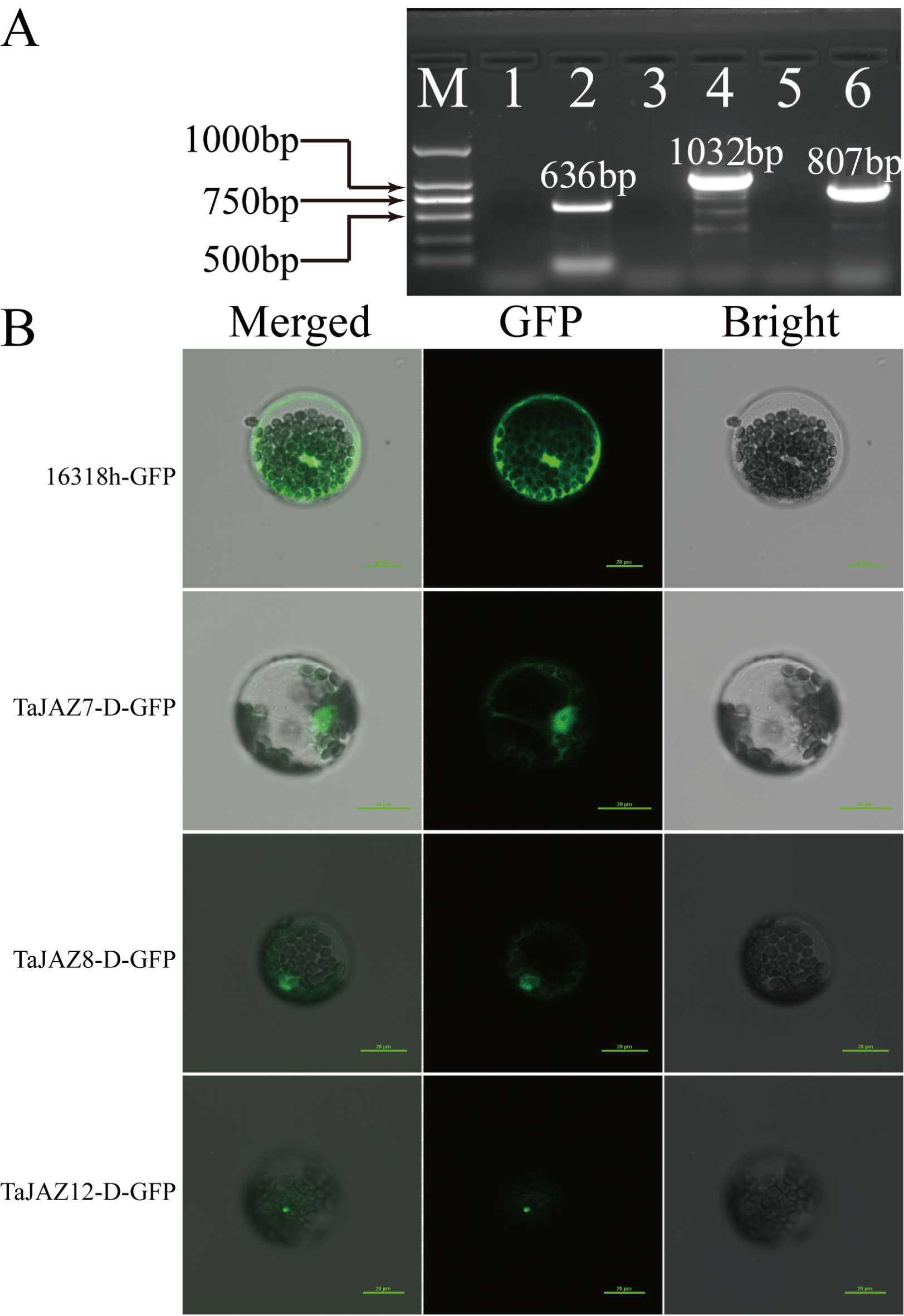

Supplement: Additional file 1: — Figure S1. The multiple sequence alignment of TaJAZ proteins. The pink and green lines represent the core sequence of TIFY domain and Jas domain in each of TaJAZ protein. The blank boxes represent the NLS sequence in each of TaJAZ protein. The location of TIFY domain and Jas domain were also marked out in each of TaJAZ protein. Figure S2. Phylogenetic relationship of JAZ between B. distachyond and wheat. The BdJAZs in the indicated groups were used as an outgroup to calculate Ka and Ks. Figure S3. The PCR amplified products and subcellular location of TaJAZ7-D, TaJAZ8-D and TaJAZ12-D. (A) lane 1, 3 and 5 represent negative control;. lane 2: the PCR amplified product of TaJAZ7-D; lane 4: the PCR amplified product of TaJAZ8-D; lane 6: the PCR amplified product of TaJAZ12-D. (B) the subcellular locations of TaJAZ7-D-GFP, TaJAZ8-D-GFP and TaJAZ12-D-GFP fusion proteins. Scale bars = 20 μm. (ZIP 3354 kb) [file 12864_2017_3582_MOESM1_ESM.zip › Figure S3.tif]
