# Supplementary material for: Genome-wide characterization of JASMONATE-ZIM DOMAIN transcription repressors in wheat (Triticum aestivum L.)
Source: BMC Genomics. 2017 Feb 13;18:152. doi: 10.1186/s12864-017-3582-0 (PMC5307646; doi:10.1186/s12864-017-3582-0)
Supplement: Additional file 2: — Table S1. The primers used in this study. Table S2. The information of JAZ or JAZ-like genes in different plants. Table S3. The number and composition of cis- acting regulatory elementsof each TaJAZ gene. (ZIP 40 kb) [file 12864_2017_3582_MOESM2_ESM.zip › Table S1.doc]

Table S1 The primers used in this study

| Primer name | Forward sequence | Reverse sequence | Annotation |
| --- | --- | --- | --- |
| TaJAZ1-RT | CAGGAAGAAGGAGACCATGGAG | CAGCACGGCCGTCTGGA | qRT-PCR analysis |
| TaJAZ2-RT | GCAGCAGCAGCTGCGG | AGCCTGAAGCTTGCAAATCG |  |
| TaJAZ3-RT | TCCGAGGGATGGAGACGAG | GGAGCCGTTGTAGAAGACCGT |  |
| TaJAZ4-RT | GAAATTCCACCCGGTCTATCTG | TCTTTGCGTACAGCATAGCGTAT |  |
| TaJAZ5-RT | CACCACGCTCAGCCTCATG | ACCCTGCCGCCGTAGAA |  |
| TaJAZ6-RT | ACCACGCTCAGCCTCATGCCC | CCACCCTGCCGCCGTAGAAGA |  |
| TaJAZ7-RT | ATGGGAGCCGAGCAGGA | CTCGTGCGAGCCCCTGA |  |
| TaJAZ8-RT | CCGCTAAGTAATGCACCCTCA | AACTCTGTCTTTCCGCTTCTCC |  |
| TaJAZ9-RT | TGACAACTTCCCGTCCACCAA | CGCCACTATCCTGCCCTTTCT |  |
| TaJAZ10-RT | TGCACCCACTACTGTTACTCGC | GGAGACATGCAGCTTGAGACAT |  |
| TaJAZ11-RT | AAAAGCGCAAGGAAAGGAAC | CTGGCATACAGGTGCAGAAA |  |
| TaJAZ12-RT | ATGGAGATGGACTTTCTGGGC | CACGGTGGACTACCTGCG |  |
| TaJAZ13-RT | TTCTACGGCGGGCAGGTGCT | GAGCCTTCCCTTGCGCTTCG |  |
| TaJAZ14-RT | TAGGGAGAAGCGGAAAGAGC | TCCACGACGCATCATAGGTG |  |
| TaActin-RT | TACTCCCTCACAACAACCG | AGAACCTCCACTGAGAACAA | Reference gene for qRT-PCR |
| TaJAZ7-D-ORF | ATGGGAGCCGAGCAGCAGCAGC | TCAGTTGAGCTGGAGCGCGGAGTTG | Cloning of *TaJAZ7-D*, *8-D* and *12-D* |
| TaJAZ8-D-ORF | ATGAAGCCGGACTCAGAATACC | TTATTCCAACTCAATAACCACCCTA |  |
| TaJAZ12-D-ORF | ATGGAGATGGACTTTCTGGGC | CTATATCTTGAGCTCCGTGTCCAG |  |
| TaJAZ7-D-SL | GCGTCGACATGGGAGCCGAGCAGCAGCA | GCTCTAGAGTTGAGCTGGAGCGCGGAGTTG | Subcellular location of TaJAZ7-D-GFP, 8-D-GFP and 12-D-GFP fusion proteins |
| TaJAZ8-D-SL | GCGTCGACATGAAGCCGGACTCAGAATACC | GCTCTAGATTCCAACTCAATAACCACCCTACG |  |
| TaJAZ12-D-SL | GCGTCGACATGGAGATGGACTTTCTGGGC | GCTCTAGATATCTTGAGCTCCGTGTCCAG |  |
