# Supplementary material for: Genome-wide characterization of JASMONATE-ZIM DOMAIN transcription repressors in wheat (Triticum aestivum L.)
Source: BMC Genomics. 2017 Feb 13;18:152. doi: 10.1186/s12864-017-3582-0 (PMC5307646; doi:10.1186/s12864-017-3582-0)
Supplement: Additional file 2: — Table S1. The primers used in this study. Table S2. The information of JAZ or JAZ-like genes in different plants. Table S3. The number and composition of cis- acting regulatory elementsof each TaJAZ gene. (ZIP 40 kb) [file 12864_2017_3582_MOESM2_ESM.zip › Table S2.doc]

Table S2 The information of *JAZ* or *JAZ*-like genes in different plants

| Species | Locus name/Accession number | Gene name | A.A length |
| --- | --- | --- | --- |
| *Physcomitrella patens* | Pp1s15_168V6.1 | *PpJAZ1* | 391 |
|  | Pp1s88_112V6.1 | *PpJAZ2* | 441 |
|  | Pp1s88_114V6.1 | *PpJAZ3* | 500 |
|  | Pp1s103_27V6.1 | *PpJAZ4* | 482 |
|  | Pp1s103_29V6.2 | *PpJAZ5* | 297 |
|  | Pp1s442_10V6.1 | *PpJAZ6* | 502 |
|  | Pp1s442_14V6.1 | *PpJAZ7* | 498 |
| *Selaginella moellendorffii* | estExt_fgenesh2_pg.C_60080 | *SmJAZ1* | 280 |
|  | estExt_fgenesh2_pg.C_1190037 | *SmJAZ2* | 441 |
|  | fgenesh2_pg.C_scaffold_26000244 | *SmJAZ3* | 235 |
|  | fgenesh2_pg.C_scaffold_40000122 | *SmJAZ4* | 480 |
|  | fgenesh2_pg.C_scaffold_87000129 | *SmJAZ5* | 1346 |
|  | estExt_fgenesh2_pg.C_530065 | *SmJAZ6* | 311 |
| *Picea sitchensis* | ABR16076.1 | *PsJAZ1* | 353 |
|  | ABR16381.1 | *PsJAZ2* | 484 |
|  | ABR17591.1 | *PsJAZ3* | 526 |
|  | ABR18272.1 | *PsJAZ4* | 260 |
|  | ABK22803.1 | *PsJAZ5* | 269 |
|  | ABK23510.1 | *PsJAZ6* | 268 |
|  | ABK25156.1 | *PsJAZ7* | 220 |
|  | ABK26593.1 | *PsJAZ8* | 286 |
|  | ABK26702.1 | *PsJAZ9* | 279 |
|  | ABK26895.1 | *PsJAZ10* | 269 |
|  | ADE76943.1 | *PsJAZ11* | 189 |
|  | ADE77031.1 | *PsJAZ12* | 401 |
|  | ADE77685.1 | *PsJAZ13* | 319 |
| *Oryza sativa* | LOC_Os03g08310.1 | *OsJAZ1* | 179 |
|  | LOC_Os03g08320.1 | *OsJAZ2* | 209 |
|  | LOC_Os03g08330.1 | *OsJAZ3* | 187 |
|  | LOC_Os03g27900.1 | *OsJAZ4* | 202 |
|  | LOC_Os03g28940.1 | *OsJAZ5* | 228 |
|  | LOC_Os04g32480.1 | *OsJAZ6* | 188 |
|  | LOC_Os04g55920.2 | *OsJAZ7* | 216 |
|  | LOC_Os07g05830.1 | *OsJAZ8* | 185 |
|  | LOC_Os07g42370.1 | *OsJAZ9* | 244 |
|  | LOC_Os08g33160.1 | *OsJAZ10* | 432 |
|  | LOC_Os09g23660.1 | *OsJAZ11* | 416 |
|  | LOC_Os09g26780.1 | *OsJAZ12* | 232 |
|  | LOC_Os10g25230.1 | *OsJAZ13* | 166 |
|  | LOC_Os10g25250.1 | *OsJAZ14* | 330 |
|  | LOC_Os10g25290.1 | *OsJAZ15* | 171 |

**Table S2 The information of JAZ or JAZ-like genes in different plants (*Continued*)**

| Species | Locus name/Accession number | Gene name | A.A length |
| --- | --- | --- | --- |
| *Sorghum bicolor* | SB01G023300 | *SbJAZ1* | 168 |
|  | SB01G023290 | *SbJAZ2* | 226 |
|  | SB01G023320 | *SbJAZ3* | 175 |
|  | SB01G023330 | *SbJAZ4* | 186 |
|  | SB01G023431 | *SbJAZ5* | 134 |
|  | SB01G027325 | *SbJAZ6* | 145 |
|  | SB01G033020 | *SbJAZ7* | 228 |
|  | SB01G045180 | *SbJAZ8* | 201 |
|  | SB01G045190 | *SbJAZ9* | 180 |
|  | SB02G003130 | *SbJAZ10* | 242 |
|  | SB02G024270 | *SbJAZ11* | 408 |
|  | SB02G025720 | *SbJAZ12* | 221 |
|  | SB02G039190 | *SbJAZ13* | 235 |
|  | SB03G044485 | *SbJAZ14* | 100 |
|  | SB06G031060 | *SbJAZ15* | 274 |
| *Arabidopsis thaliana* | AT1G19180.1 | *AtJAZ1* | 253 |
|  | AT1G74950.1 | *AtJAZ2* | 249 |
|  | AT3G17860.1 | *AtJAZ3* | 352 |
|  | AT1G48500.1 | *AtJAZ4* | 310 |
|  | AT1G17380.1 | *AtJAZ5* | 274 |
|  | AT1G72450.1 | *AtJAZ6* | 269 |
|  | AT2G34600.1 | *AtJAZ7* | 148 |
|  | AT1G30135.1 | *AtJAZ8* | 131 |
|  | AT1G70700.1 | *AtJAZ9* | 267 |
|  | AT5G13220.1 | *AtJAZ10* | 167 |
|  | AT3G43440.1 | *AtJAZ11* | 152 |
|  | AT5G20900.1 | *AtJAZ12* | 187 |
| *Brachypodium distachyon* | Bradi5g24410 | *BdJAZ1* | 196 |
|  | Bradi1g58490 | *BdJAZ2* | 168 |
|  | Bradi3g36380 | *BdJAZ3* | 433 |
|  | Bradi4g29830 | *BdJAZ4* | 418 |
|  | Bradi5g08650 | *BdJAZ5* | 172 |
|  | Bradi3g56940 | *BdJAZ6* | 221 |
|  | Bradi3g10820 | *BdJAZ7* | 235 |
|  | Bradi1g21490 | *BdJAZ8* | 237 |
|  | Bradi4g31240 | *BdJAZ9* | 244 |
|  | Bradi1g72610 | *BdJAZ10* | 179 |
|  | Bradi1g72590 | *BdJAZ11* | 188 |
|  | Bradi1g72600 | *BdJAZ12* | 204 |
|  | Bradi3g23220 | *BdJAZ13* | 224 |
|  | Bradi3g23180 | *BdJAZ14* | 150 |
|  | Bradi3g23190 | *BdJAZ15* | 146 |

Table S2 The information of JAZ or JAZ-like genes in different plants (*Continued*)

| Species | Locus name/Accession number | Gene name | A.A length |
| --- | --- | --- | --- |
| *Aegliops tauschii* | Scaffold4854:3946:40410 | *AetJAZ1* | 230 |
|  | Scaffold19622:60754:65432 | *AetJAZ2* | 245 |
|  | Scaffold32076:15816:16884 | *AetJAZ3* | 264 |
|  | Scaffold126383:3932:5440 | *AetJAZ4* | 170 |
|  | Scaffold999:111353:111781 | *AetJAZ5* | 186 |
|  | Scaffold23145:20809:21252 | *AetJAZ6* | 347 |
|  | Scaffold999:135137:136039 | *AetJAZ7* | 213 |
|  | Scaffold5772:40046:40468 | *AetJAZ8* | 139 |
| *Zea mays* | GRMZM2G343157 | *ZmJAZ1* | 233 |
|  | GRMZM2G445634 | *ZmJAZ2* | 218 |
|  | GRMZM2G117513 | *ZmJAZ3* | 404 |
|  | GRMZM2G024680 | *ZmJAZ4* | 198 |
|  | GRMZM2G145412 | *ZmJAZ5* | 160 |
|  | GRMZM2G145458 | *ZmJAZ6* | 155 |
| *Hevea brasiliensis* | GQ369508.1 | *HvJAZ1* | 284 |
|  | KJ001643.1 | *HvJAZ2* | 265 |
|  | KJ001644.1 | *HvJAZ7* | 141 |
|  | KJ001645.1 | *HvJAZ8* | 128 |
|  | KJ001646.1 | *HvJAZ9* | 381 |
|  | KJ001647.1 | *HvJAZ10* | 192 |
|  | KJ001648.1 | *HvJAZ11* | 225 |
