# Supplementary material for: Genome-wide characterization of JASMONATE-ZIM DOMAIN transcription repressors in wheat (Triticum aestivum L.)
Source: BMC Genomics. 2017 Feb 13;18:152. doi: 10.1186/s12864-017-3582-0 (PMC5307646; doi:10.1186/s12864-017-3582-0)
Supplement: Additional file 2: — Table S1. The primers used in this study. Table S2. The information of JAZ or JAZ-like genes in different plants. Table S3. The number and composition of cis- acting regulatory elementsof each TaJAZ gene. (ZIP 40 kb) [file 12864_2017_3582_MOESM2_ESM.zip › Table S3.doc]

| **Element name** | TaJAZ1 | | TaJAZ2 | TaJAZ3 | | TaJAZ4 | | | TaJAZ5 | | | TaJAZ6 | TaJAZ7 | | TaJAZ8 | TaJAZ9 | | TaJAZ10 | TaJAZ12 | | TaJAZ13 | | TaJAZ14 | **Annotation** |
| --- | --- | --- | --- | --- | --- | --- | --- | --- | --- | --- | --- | --- | --- | --- | --- | --- | --- | --- | --- | --- | --- | --- | --- | --- |
|  | 1A | 1B | 2B | 3A | 3D | 4A | 4B | 4D | 5A | 5B | 5D | 6D | 7A | 7D | 8D | 9B | 9D | 10B | 12A | 12D | 13A | 13D_2_ | 14D |  |
| ABRE | 2 | 2 | 3 | 2 | 3 |  |  |  | 4 | 4 | 4 | 2 | 6 | 2 |  | 1 | 1 | 4 |  |  | 2 | 1 | 2 | cis-acting element involved in ABA responsiveness |
| TGA-element | 2 |  |  |  |  |  | 1 |  |  |  | 2 |  |  | 1 | 2 |  |  |  | 1 |  |  | 1 |  | Auxin-responsive element |
| Box-w1 |  | 2 |  |  | 2 |  |  | 1 |  | 1 | 1 |  |  |  |  |  | 2 | 2 |  | 1 |  |  | 1 | Fungal elicitor responsive element |
| CGTCA-motif |  | 1 |  |  | 1 |  |  |  | 2 | 3 | 3 |  | 1 | 2 | 4 | 3 | 1 | 3 | 2 | 3 | 2 |  | 1 | cis-acting element involved in MeJA-responsiveness |
| GARE-motif |  |  | 1 | 1 |  | 1 | 1 | 2 |  | 1 | 1 |  | 1 | 1 |  |  |  |  | 1 | 2 |  |  |  | Gibberellin-responsive element |
| MBS |  |  | 4 |  |  |  | 2 |  |  |  |  | 2 |  |  |  | 2 | 1 | 1 |  | 2 | 3 | 2 |  | MYB binding site involved in drought-inducibility |
| LTR |  |  |  | 1 |  |  | 1 | 1 |  | 1 |  |  | 1 | 3 |  |  |  | 2 |  | 1 | 1 | 3 | 1 | low-temperature responsiveness |
| TCA-element |  |  | 1 | 2 | 3 | 1 | 2 | 1 |  |  |  |  | 2 | 2 | 2 |  |  | 3 |  |  |  | 2 |  | Salicylic acid responsive element |

Table S3 The number and composition of *cis*- acting regulatory elements of each *TaJAZ* gene
